# Efficacy of meglumine antimoniate treatment on boxer *Leishmania infantum* skin lesions: case report

**DOI:** 10.3389/fvets.2025.1600004

**Published:** 2025-06-30

**Authors:** Cristina Carresi, Clara Francesca Ferrucci, Cyndi Mangano, Anna Rita Coppoletta, Antonio Cardamone, Vincenzo Musolino, Micaela Gliozzi, Vincenzo Mollace, Domenico Britti

**Affiliations:** ^1^Pharmacology Laboratory, Department of Health Sciences, Interregional Research Center for Food Safety and Health IRC-FSH, University “Magna Graecia” of Catanzaro, Catanzaro, Italy; ^2^Veterinary Clinic “Tripodi”, Reggio Calabria, Italy; ^3^Pharmaceutical Biology Laboratory, Department of Health Sciences, Interregional Research Center for Food Safety and Health IRC-FSH, University “Magna Graecia” of Catanzaro, Catanzaro, Italy; ^4^Department of Health Sciences, University “Magna Graecia” of Catanzaro, Catanzaro, Italy

**Keywords:** *Leyshmania infantum*, meglumine antimoniate, boxer, cutaneous leishmaniasis, skin lesions, subcutaneous injections

## Abstract

This clinical report describes the beneficial effects of local subcutaneous injections of meglumine antimoniate (Glucantime®) on *Leishmania* cutaneous lesions in a dog from Calabria, a region of Southern Italy. Leishmaniasis is an endemic zoonotic disease in the European Union, particularly in Mediterranean countries, as well as in parts of north and east Africa, India, China, and Central and South America, caused by protozoa of the genus *Leishmania* spp., which infect several reservoirs, including humans and domestic animals. In southern Europe, the main etiological agent is *Leishmania infantum,* transmitted by sandflies of the subfamily Phlebotominae, which is the most common cause of cutaneous leishmaniasis (CL) in these regions, where dogs are considered the primary domestic reservoir of the parasite. A 7-year-old male non-sterilized Boxer named Ettore underwent pre-vaccination blood tests and Leishmania indirect immunofluorescence (IFI) test, which confirmed the presence of antibodies against the protozoan *Leishmania infantum* (antibody titer, 1:1280), supporting the diagnosis of CL. The dog underwent a therapeutic protocol consisting of miltefosine (Milteforan™ - Virbac®) (2 mg/Kg b.w. per os) for 28 days and allopurinol 300 mg (10 mg/Kg b.w. po) for 6 months. However, at the end of the treatment period, the appearance of a suspicious skin lesion on the left tarsus was reported, which appeared inflamed and infected. The subsequent antibiotic and anti-inflammatory therapy based on amoxicillin+clavulanic acid (12.5 mg/kg b.w. po for 15 days), metronidazole (75000UI + 12.5 mg po for 15 days), and prednisone (0.5 mg/kg b.w. po for 10 days) failed to be effective; thus, the lesion worsened and also spread to the dorsal femoral surface of both hind limbs, presenting as blackish, swollen, painful, alopecic and oozing bloody and purulent material. Mild renal microlithiasis and splenopathy were reported by abdominal ultrasound and were associated with a possible leishmania pattern. Finally, skin lesions were experimentally treated with subcutaneous injections of Glucantime® (200 mg/lesion – 0.5 mL/lesion) once a month for 5 months, followed by complete healing. Interestingly, the experimental localized treatment with Glucantime® proved to be crucial in counteracting Leishmania skin lesions. The results obtained suggest that, through an appropriate diagnosis, it is possible to define targeted and effective therapeutic protocols useful in the management of canine leishmaniasis.

## Introduction

Leishmaniasis is an endemic zoonotic disease in part of the European Union, particularly in Mediterranean countries, caused by protozoa of the genus *Leishmania* spp., which have a variety of reservoirs, including humans and domestic animals (1). There are several species of Leishmania (including *Leishmania infantum*, *Leishmania major*, *Leishmania tropica*, and *Leishmania donovani*) that can cause different forms of the disease, characterized by a range of symptoms from mild to severe, depending on the *Leishmania* species involved and the host’s immune response (2). Among the most important zoonotic diseases, leishmaniasis is considered the third most important vector-borne disease after malaria and filariasis, representing a major concern for public health (3, 4).

The epidemiology of leishmaniases is dynamic, and the conditions of transmission are continually evolving due to climatic and environmental changes, the migration of infected people and animal reservoirs, human behavior, socioeconomic status, and immunogenic profile of affected human populations (5, 6). The clinical spectrum of leishmaniasis includes focal cutaneous leishmaniasis (CL) with single or multiple skin ulcers; satellite lesions or nodular lymphangitis; mucocutaneous (MCL), with skin lesions involving the mucosa; and disseminated visceral leishmaniasis (VL), generally without skin involvement and affecting internal organs, such as liver, spleen, and bone marrow (7–9). VL, caused by *Leishmania donovani* in Asia and Africa and by *Leishmania infantum* in the Mediterranean Basin, the Middle East, Central Asia, South America, and Central America, represents a life-threatening disease affecting ≈200,000–400,000 persons annually and causes an estimated ≈20,000–40,000 deaths per year (10, 11).

Although the number of reported cases has decreased over the last decade due to improved access to diagnosis and treatment and more intensive vector control, WHO defines leishmaniasis as a category 1 emerging and uncontrolled disease and lists it as one of the “neglected” tropical diseases for which the development of new treatments is a priority (12, 13). In southern Europe, the main etiological agent is *Leishmania infantum,* which is transmitted by sandflies of the subfamily Phlebotominae, which represents the most frequent cause of CL. In these regions, leishmaniasis is subclinical in humans and emerges preferentially in immunosuppressed individuals. In contrast, leishmaniasis in domestic animals has a higher incidence, and dogs are considered the chief domestic reservoir of the parasite (14, 15).

From a veterinary perspective, it is estimated that over 700 million dogs worldwide are infected, with the vast majority living in close contact with humans (16).

A recent study has well summarized the pooled global prevalence of *Leishmania infantum* infection in dogs over the last three decades. Estimating the global prevalence, subgrouping by continent, country, diagnostic test, and selected risk factors, is crucial to explain the differences between the estimated prevalence obtained (17).

.The pooled global prevalence obtained from the random-effects meta-analysis was 15.2% (95%CI: 13.6–16.9), mostly in rural (19.5%) and owned dogs (16.5%), which varies slightly depending on the diagnostic test performed (17).

A sub-group analysis performed for continents revealed the highest values of prevalence in South and Central America (18%), followed by Africa (17%), Europe (16%), and Asia (11%) (17).

Other epidemiological surveys found positive dogs in 36 countries, and the pooled prevalence ranged between 4 and 21% in European countries, between 12 and 31% in South and Central American countries, between 2 and 33% in Asian countries, and between 3 and 27% in African countries. These results reflect heterogeneity in the continents’ prevalence, the highly variable dynamics of vector ecology, and the spread and redistribution of vector-borne diseases, which are strongly influenced by environmental factors and climate change (18). Other recent investigations reported that the prevalence of affected dogs reached 30–40% in Mediterranean regional populations (19). An interesting survey performed using PCR and immunoblotting techniques or serological and cell-mediated tests found *Leishmania* infection positivity rates of 53.1, 65, and 80% in asymptomatic dogs living in Italy, France, and Portugal, respectively (20–22). Furthermore, a large population of *Leishmania*-infected dogs identified in endemic regions appear almost clinically healthy but remain carriers of the parasite, serving as a source for phlebotomine vector sandflies and playing an active role in the transmission of *Leishmania* (23, 24).

Prevalence rates of *Leishmania infantum* were also calculated based on recognized risk factors such as population type (stray, owned, and shelter dogs) and urbanization (dogs from rural and urban areas), demonstrating the highest prevalence of infection in owned dogs (18%) compared to stray (9%) and shelter dogs (15%) and in dogs living in rural areas (20%) compared to those living in urbanized (13%) or mixed (14%) areas, highlighting the risk of human Leishmaniosis (17, 25, 26). In dogs, *Leishmania infantum* causes a broad spectrum of clinical signs that vary greatly from asymptomatic/mild to severe disease due to the parasite’s spread in the skin and internal organs, immunological status, and genetic background of the host (27).

Despite the viscerotropic nature of *Leishmania infantum*, skin lesions are the most frequent manifestation of canine leishmaniasis.

The CL disease in dogs is characterized by local, ulcerative lesions on the ears, scrotum, feet, nipples, and muzzle. Skin lesions are often associated with generalized dermatitis, alopecia, amentaceous dandruff, and lymphadenopathy. Typically, affected dogs present with a poor body condition and often exhibit weight loss. In addition, auricle vascular lesions and focal dermatitis are commonly observed (27–29).

Early diagnosis and effective treatment of canine leishmaniasis cases are crucial for the control of the zoonotic cycle and help prevent the spread of the disease to other animals and humans, which remains a challenge (30).

For over 60 years, the use of pentavalent antimonials has been the main therapeutic option for the treatment of leishmaniasis and is still used in recent times in the form of methylglucamine antimoniate or sodium stibogluconate administered by intralesional, intramuscular, or intravenous injections (31, 32).

Pentavalent antimonials are prodrugs metabolized to trivalent antimony, the active molecule, whose action mechanism is involved in the inhibition of energy metabolism and macromolecular biosynthesis via glycolysis inhibition, fatty acid *β*-oxidation, and DNA damage, although it is not yet fully known (33).

However, several studies have reported clinical adverse events associated with pentavalent antimonial treatment in infected dogs, with the occurrence of adverse events, including local, systemic, and idiosyncratic skin reactions as well as a potential negative impact on renal function, especially if already compromised (34, 35). The most frequently reported clinical events include apathy, anorexia, vomiting, diarrhea, and pain at the injection site, but hepatotoxicity, acute pancreatitis, and up to severe renal failure may also occur (36–39).

Furthermore, cases of antimony resistance in canine leishmaniasis were reported, and the spread of antimony-resistant strains represents a concrete issue, given the widespread use of meglumine antimoniate in dogs and a possible non-rational use of this drug in veterinary practice (40).

In recent times, in addition to pentavalent antimonial, the main chemotherapeutic options include liposomal amphotericin B, miltefosine, paromomycin, and pentamidine (41).

Over the last 15 years, miltefosine has been increasingly used by veterinarians with some undeniable advantages, such as daily oral administration (compared to subcutaneous administration of pentavalent antimonial) and mild adverse effects, that are mostly limited to dose-dependent gastrointestinal reactions (42). Miltefosine represents the first and only oral FDA-approved antileishmanial drug [Paladin Therapeutics (2014). Impavido Package, Reference ID: 3473184]. The US Food and Drug Administration (FDA) is used to treat VL, CL, and kala-azar dermal leishmaniasis, and it is also currently in clinical development as part of combination therapy regimens (11, 43). Its mechanism of action is associated with lipid binding and apoptosis triggering (44).

The first-choice treatments used for controlling canine leishmaniasis consist of various dosage regimens of leishmanicides (pentavalent antimonial or miltefosine) combined with allopurinol (a leishmanistatic drug) (37, 45, 46), which primarily target intracellular amastigotes in tissue macrophages (33, 47). Additionally, immune therapy (e.g., domperidone) and dietary nucleotide administration with a hexose-related active compound are effective in reducing infectivity (48, 49).

The purpose of these pharmacological treatments is to control clinical signs and hematobiochemical alterations, improve the dog’s cellular immunity, reduce the parasitic load, prevent relapses, and decrease the transmission rate to the vector, leading to parasitological clearance (50–52). However, this goal is not often achieved, and remission may only be temporary, and clinical relapses can occur (33, 36, 53). Treatment failures were observed in monotherapy and combined therapies (54, 55), with improvement in canine symptomatology not always followed by parasitological clearance (56).

Therefore, existing pharmacological treatments are not yet considered a fully effective control measure, both because the probability of relapses is frequent and dogs may continue to be infectious despite being clinically cured (57) and because of the risk that the parasite will develop resistance and cross-resistance mechanisms (58, 59).

Hence, the implementation of leishmaniasis prevention programs based on the limitation of reservoirs through screening, vaccination, and the control of sand fly bites with the regular use of topical repellent insecticides is mandatory (60).

Furthermore, there is a clear need to improve the application of current pharmacological treatments by developing targeted therapeutic protocols useful for improving the treatment efficacy, reducing toxicity, and minimizing the consequent occurrence of side effects often caused by parenteral administration.

Intralesional pentavalent antimonial has been used for decades to treat human CL in the Old World.

Intralesional treatment with meglumine antimoniate was introduced in Rio de Janeiro in the 1980s using a method developed and implemented at the Evandro Chagas National Institute of Infectious Diseases (NIID) (61–63) and subsequently widely applied, especially in patients with contraindications to the systemic use (64). In 2010, the World Health Organization (WHO) recommended the use of safer and less toxic lesion-targeted treatments for the control of leishmaniasis (65, 66). More recent studies comparing systemic meglumine antimoniate therapy with intralesional ones in human patients suggested that localized treatment of CL lesions is simple, efficient, and safe (63, 67). These findings reveal how subcutaneous administration of pentavalent antimony could be a valid and effective alternative to systemic treatment, leading to the resolution of canine leishmania-induced skin lesions.

Thus, the present clinical report aims to assess the possible beneficial effects of experimental local subcutaneous injections of meglumine antimoniate (Glucantime®) on Boxer *Leishmania infantum* skin lesions, demonstrating how localized treatment of *Leishmania* lesions could be more effective and less toxic than systemic treatment.

## Case description

### Case presentation, diagnostic assessment, therapeutic intervention, follow-up, and outcomes

A 7-year-old male non-sterilized client-owned Boxer underwent pre-vaccination blood tests and *Leishmania* indirect immunofluorescence (IFI) test. Historically, the dog’s vaccination status and flea and ticks prevention measures were up to date, including the use of a collar (Seresto® Elanco) impregnated with imidacloprid 4.5 g + flumetrina 2.03 g. On physical examination, the dog was afebrile (rectal temperature, 38.28°C), with a heart rate of 96 beats/min, a respiratory rate of 24 breaths/min, weighed 40 kg, and was in good physical condition.

**Figure 1:**
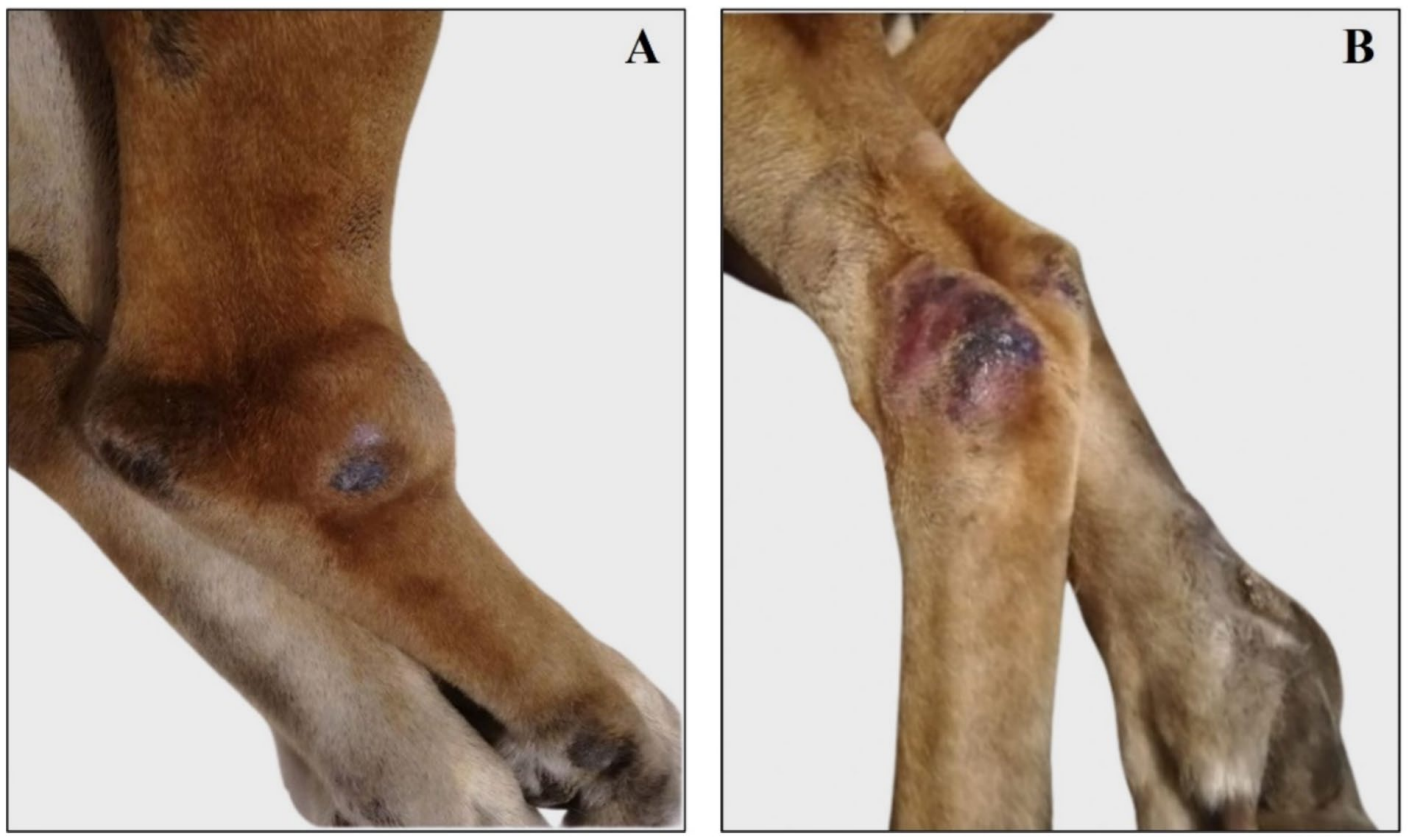
Representative images of right and left tibio-tarsal lesions reassessed after treatment with miltefosine. **(A)** Right tibio/tarsal lesion is painful and swollen. **(B)** Left tibio/tarsal lesion is alopecic and slightly swollen.

Normal hematologic analysis was observed and included a red blood cells (RBC) count, 7.40106/mm^3^ [reference range, 5.50 to 8.50106/mm^3^]; Hemoglobin (HGB), 15.4 g/dL [reference range, 15 to 20 g/dL]; haematocrit (HCT), 47.9% [reference range, 44 to 57%]; platelet (PLT), 288103/mm^3^ [reference range, 200 to 460,103/mm^3^]; mean corpuscular volume (MCV), 65 μm^3^ [reference range, 60 to 77 μm^3^]; mean cellular haemoglobin concentration (MCHC), 32.1 g/dL [reference range, 31.0 to 36.0 g/dL]; red blood cell distribution width (RDW), 14% [reference range, 14 to 17%]; mean platelet volume (MPV) 9.4 μm^3^ [reference range, 6.7 to 11.1 μm^3^]; and a slight leukopenia White blood cells (WBC), 4.7 L 103/mm^3^ [reference range, 6 to 12,103/mm^3^]. Serum biochemical analyses revealed a mildly hyperglobulinemia [serum globulin concentration, 6.2 g/dL (reference range, 2.3 to 5.2 g/dL)], a slight hyperamylasemia [2,585 U/L (reference range, 400 to 2,500 U/L)], and low albumin:globulin (A: G) ratio [0.5 g/dL (reference range, 0.59 to 1.11 g/dL)] (Table 1). Routine electrophoretic analysis revealed a gammopathy [serum *γ*-globulin concentration, 19.9% (reference range, 5 to 15%)] (Table 1). The serological investigation confirmed the presence of antibodies against protozoan *Leishmania infantum* [antibody titer, 1:1280 (antibodies titers > 1:80 are considered indicative of prior exposure or active infection)], thereby supporting the diagnosis of canine leishmaniasis (Table 1). The dog underwent the therapeutic protocol of miltefosine (Milteforan™ - Virbac®) (2 mg/Kg b.w. po) for 28 days and allopurinol 300 mg (10 mg/Kg b.w. po) for 6 months. At the end of the treatment, the dog was re-evaluated, and the routine electrophoretic analysis for *γ*-globulin test was within the normal range [serum γ-globulin concentration, 11.3% (reference range, 5 to 15%)] (Supplementary Figure 1). However, the dog was then referred due to the appearance of a suspicious non-healing skin lesion. Physical examination revealed the presence of enlarged, palpable lymph nodes (popliteal and prescapular), and skin examination revealed painful, pruritic, swollen, and alopecic cutaneous lesions on the dorsal tibio/tarsal surface of both hind limbs (Figure 1).

**Table 1:** Results of hematologic analysis, serum biochemical profile, protein electrophoresis, and indirect immunofluorescence (IFI) test for the first evaluation.

Hematologic analysis			
Exam	Result	Unit	Reference range
RBC	7.40	106/mm^3^	5.50 to 8.50
HGB	15.4	g/dL	15 to 20
HCT	47.9	%	44 to 57
PLT	288	103/mm^3^	200 to 460
MCV	65	μm^3^	60 to 77
MCHC	32.1	g/dl	31.0 to 36.0
RDW	14	%	14 to 17
MPV	9.4	μm^3^	6.7 to 11.1
WBC	**4.7**	L 103/mm^3^	6 to 12

Serum biochemical analysis			
Exam	Result	Unit	Reference range
ALB	2.8	g/dL	2.5 to 4.4
TP	9.0	g/dL	5.4 to 8.2
GLO	**6.2**	g/dL	2.3 to 5.2
Ca^2+^	10	mg/dL	8.6 to 11.8
GLU	90	mg/dL	70 to 143
BUN	12.5	mg/dL	7 to 25
P	3.97	mg/dL	2.9 to 6.6
AMY	**2,585**	U/L	400 to 2,500
CHOL	147	mg/dL	124 to 271
ALT	54	U/L	10 to 118
TBIL	0.36	mg/dL	0.1 to 0.6
ALP	61	U/L	20 to 150
CRE	1.20	mg/dL	0.3 to 1.4
BUN/CRE	10		
CK	129	U/L	90 to 440
A/G	**0,5**	g/dL	0.59 to 1.11

Electrophoretic analysis			
Exam	Result	Unit	Reference range
α-globulin	11.6	%	10 to 18
α1-globulin	5.50	%	2 to 6
α2-globulin	6.10	%	7 to 15
β-globulin	22	%	15 to 22
β1-globulin	3.60	%	2 to 5
β2-globulin	6.90	%	2 to 9
β3-globulin	12	%	5,8 to 12
γ-globulin	**19.9**	%	5 to 15
Total prot.	**8.10**	g/dL	5.5 to 7.60

IFI (Indirect immuno-fluorescence)			
Exam	Result	Unit	Reference range
Antibody titre *Leishmania infantum* (IFI) IgG	**1:1280**		1:80

RBC: red blood cells; HGB: Hemoglobin; HCT: haematocrit; PLT: platelet; MCV: mean corpuscular volume; MCHC: mean cellular haemoglobin concentration; RDW: red blood cell distribution width; MPV: mean platelet volume; WBC: White blood cells; ALB: albumin; TP: serum protein concentration; GLO: globulin; Ca2+: calcium; GLU: glucose; BUN: blood urea nitrogen; P: phosphate; AMY: amylase; CHOL: cholesterol; ALT: alanine aminotransferase; TBIL: total bilirubin; ALP: alkaline phosphatase; CRE: creatinine; CK: creatine kinase; A/G: albumin/globulin.

**Figure 2:**
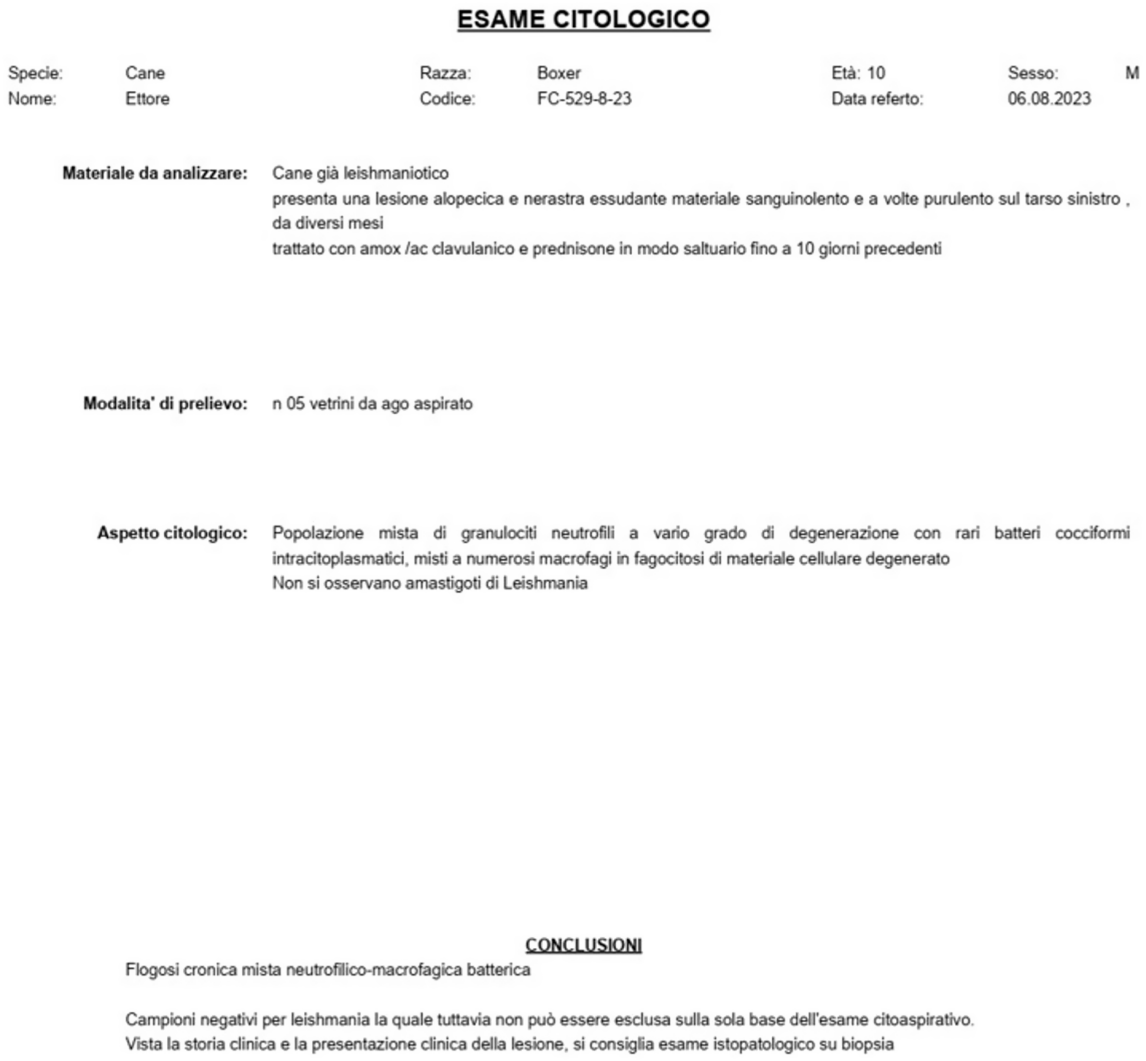
Cytological examination. Report of the cytological examination of the left tarsal lesion performed after treatment with miltefosine.

The infection was confirmed by cytological examination of the left tarsal lesion, which revealed chronic inflammation with a mixed population of neutrophilic granulocytes, varying degrees of degeneration, and rare intracytoplasmic cocci bacteria mixed with numerous macrophages. *Leishmania* amastigotes were not observed (Figure 2). The dog was treated, as needed, with amoxicillin+clavulanic acid (12.5 mg/kg b.w. po for 15 days), metronidazole (75000UI + 12.5 mg po for 15 days), and prednisone (0.5 mg/kg b.w. po for 10 days) to counteract the onset of recurrent bacterial infections, and treatment with allopurinol was resumed (10 mg/Kg b.w. po, 1 month of treatment every 6 months).

**Figure 3:**
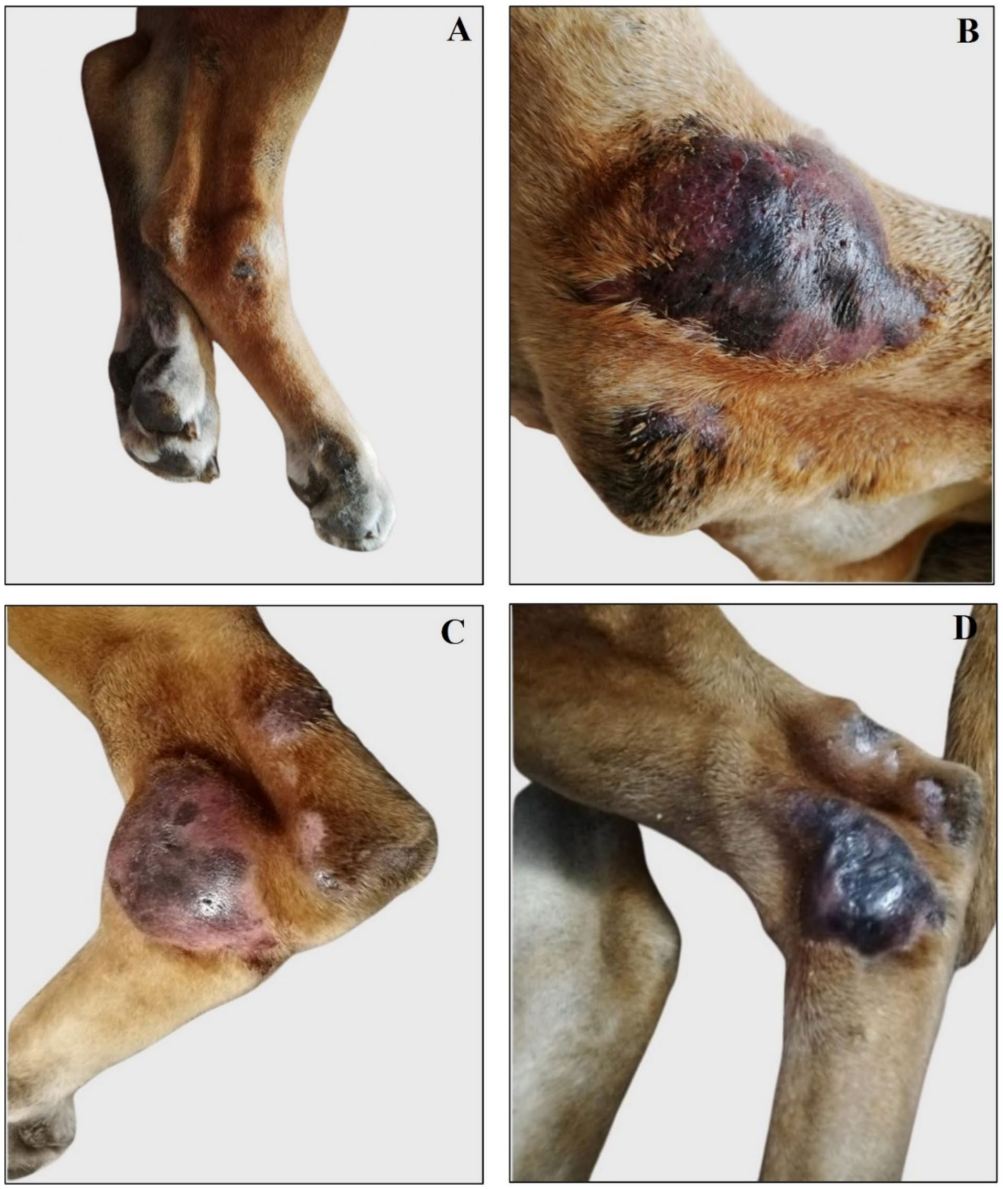
Representative images of right and left tibio/tarsal lesions reassessed during and after antibiotic therapy. **(A)** Right tibio/tarsal lesion was deflated and painless during antibiotic therapy. **(B)** Right tibio/tarsal lesion is painful, swollen, alopecic, and oozing bloody material after the end of antibiotic therapy. **(C)** Left tibio/tarsal painful, alopecic, and swollen lesion during antibiotic therapy. **(D)** Left tibio/tarsal blackish, alopecic, and deflated lesion after the end of antibiotic therapy.

**Figure 4:**
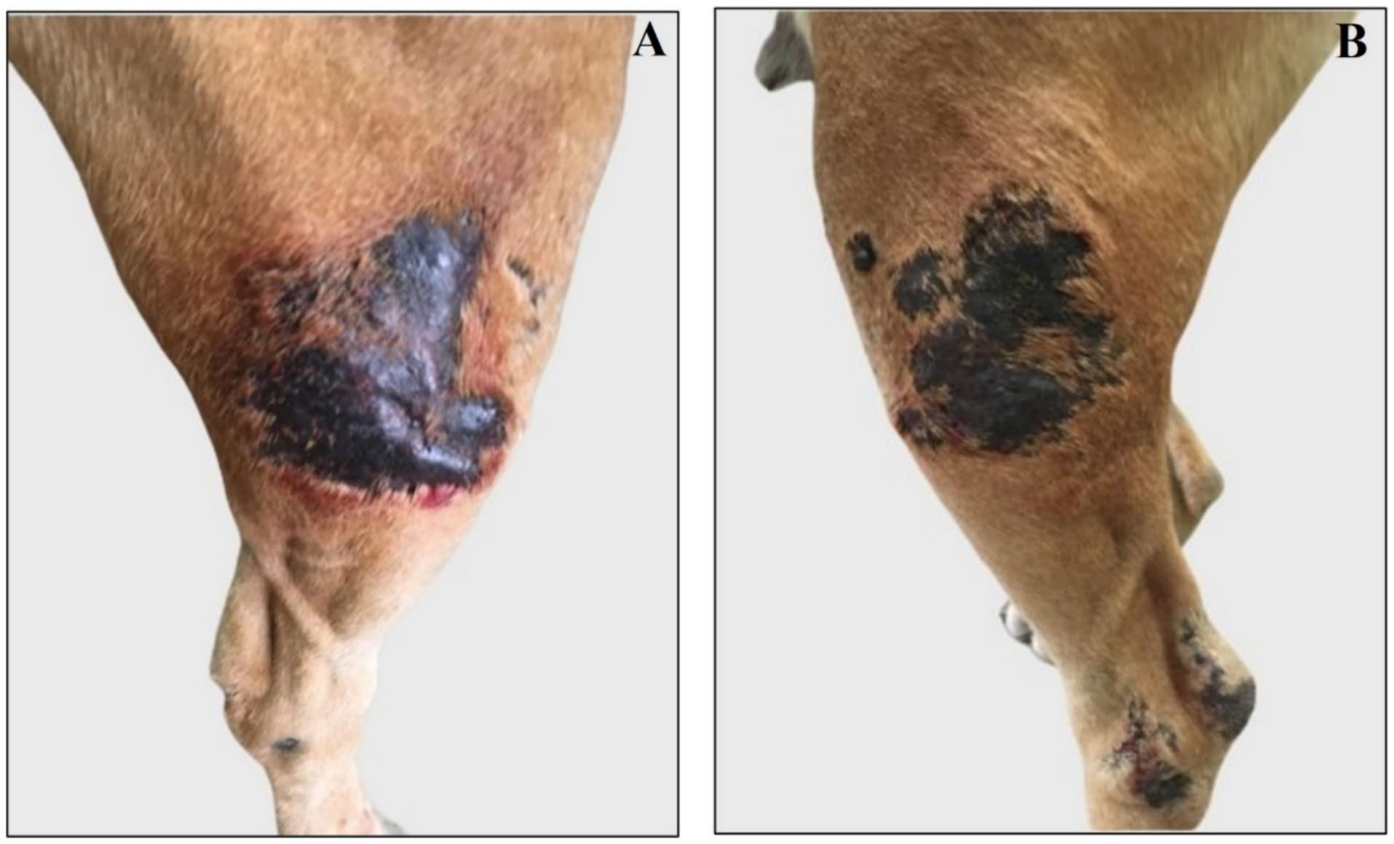
Representative images of right and left femoral new lesions assessed after antibiotic therapy. **(A)** Right femoral lesion is blackish, alopecic, and swollen. **(B)** Left femoral lesion is blackish and alopecic.

The dog was re-evaluated once a month for the next 6 months, after which the owner reported a substantial clinical decline. Physical examination revealed that the antibiotic and anti-inflammatory therapy was not effective; thus, the dorsal tibio/tarsal lesions worsened (Figure 3), and new lesions spread to the dorsal femoral surface of both hind limbs, appearing blackish, swollen, painful, alopecic, and exuding bloody and purulent material (Figure 4).

An abdominal ultrasound revealed a possible clinical pattern of a leishmanial subject, highlighting the presence of renal microlithiasis and mild splenopathy. In particular, the bladder appeared jagged with lithiasis, hyper echogenicity of the renal pillars of the left kidney was recorded, which was compatible with renal lithiasis, and the spleen increased in volume and presented a non-homogeneous parenchyma (Supplementary Figure 2). The structure, size, and vascularization of the other organs analyzed were normal (Supplementary material 1). Echocardiographic analysis showed physiological values of ejection fraction and fractional shortening, normal sinus rhythm, absence of volume and pressure overload, normal mitral and tricuspid valves, and normal aortic and pulmonary arteries (Supplementary material 2).

**Figure 5:**
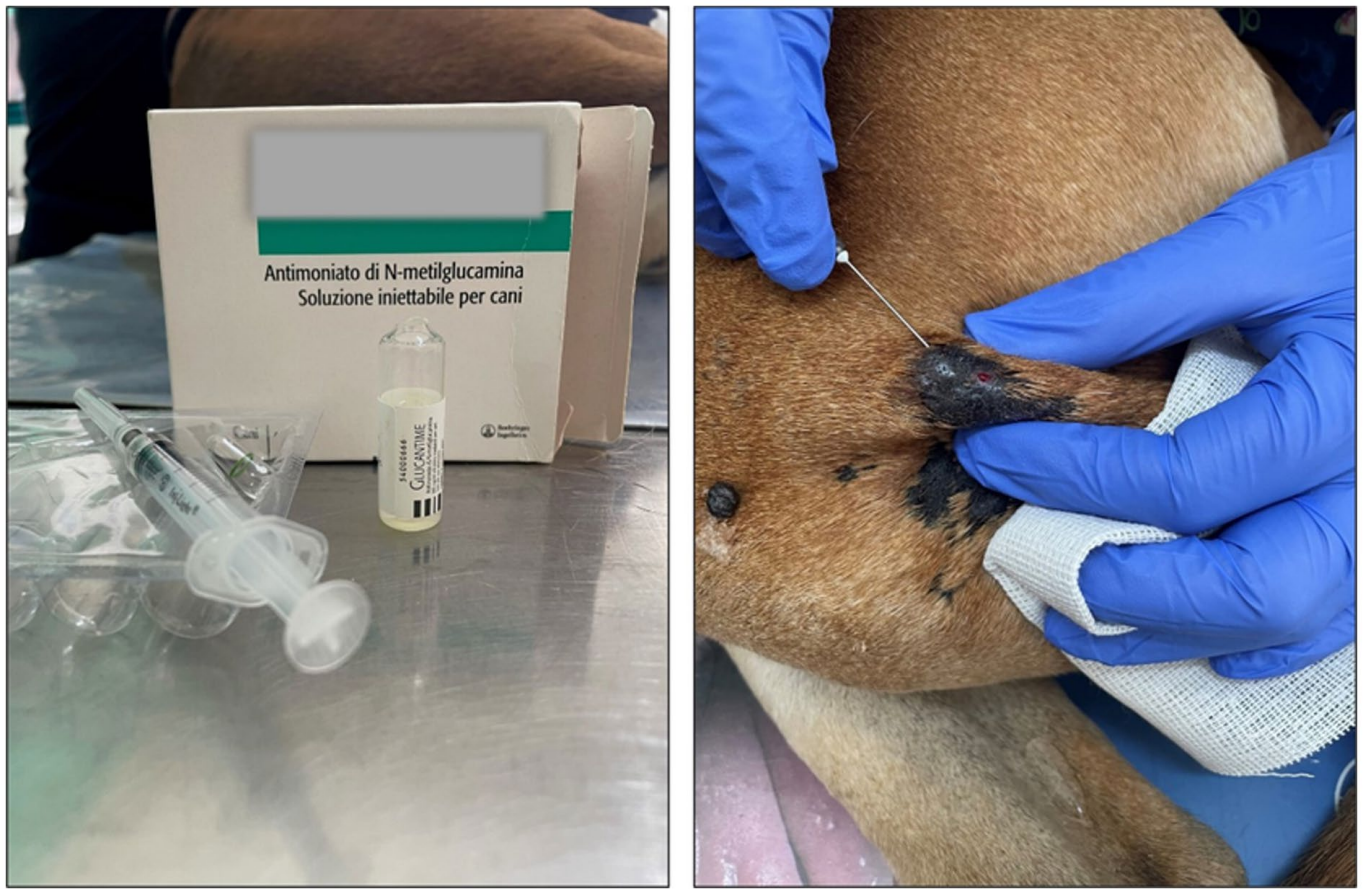
Treatment with Glucantime® (A) Vial of Glucantime® (200 mg/lesion – 0.5 mL /lesion) **(B)** Example of subcutaneous injection performed by a veterinarian along the lesion perimeter. Before the procedure, the lesion was disinfected with 10% Betadine cutaneous solution, and finally, it was swabbed with ice.

Finally, with the informed consent from the dog’s owners, skin lesions were experimentally treated with subcutaneous injections of meglumine antimoniate (Glucantime®) (200 mg/lesion – 0.5 mL /lesion) (Figure 5). The experimental treatment performed on the dog was proposed based on existing WHO indications for the treatment of human CL (58, 59).

Briefly, the dog was immobilized, and the area around each lesion was cleaned with 10% Betadine cutaneous solution. Glucantime® was injected with a needle 13 × 4.0G subcutaneously around the lesion, with the volume necessary to infiltrate the base of the lesion, leaving it raised and swollen (Figure 5). Finally, each lesion was swabbed with ice.

The application was repeated once a month for the next 5 months, periodically monitoring the progress of the lesions, behavior, and the health status of the dog.

**Figure 6:**
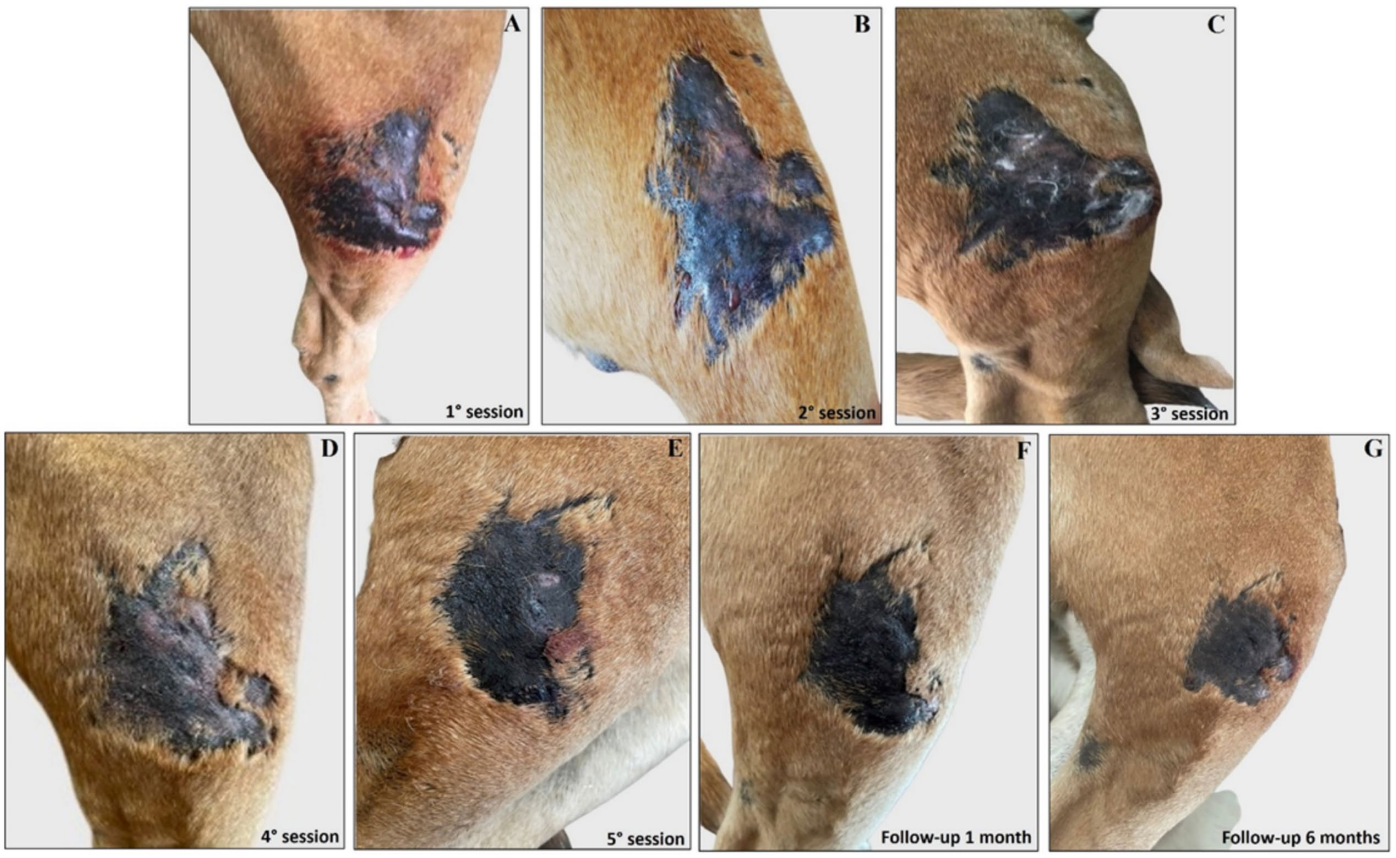
Treatment evolution of right hindlimb femoral lesions with Glucantime®. Representative images of femoral lesions on the right hindlimb during the Glucantime® experimental protocol. The images show femoral lesions before the beginning of subcutaneous injection of Glucantime® **(A)**, before every subsequent session **(B–E)**, and at 1 month **(F)** and 6 months of follow-up **(G)**.

**Figure 7:**
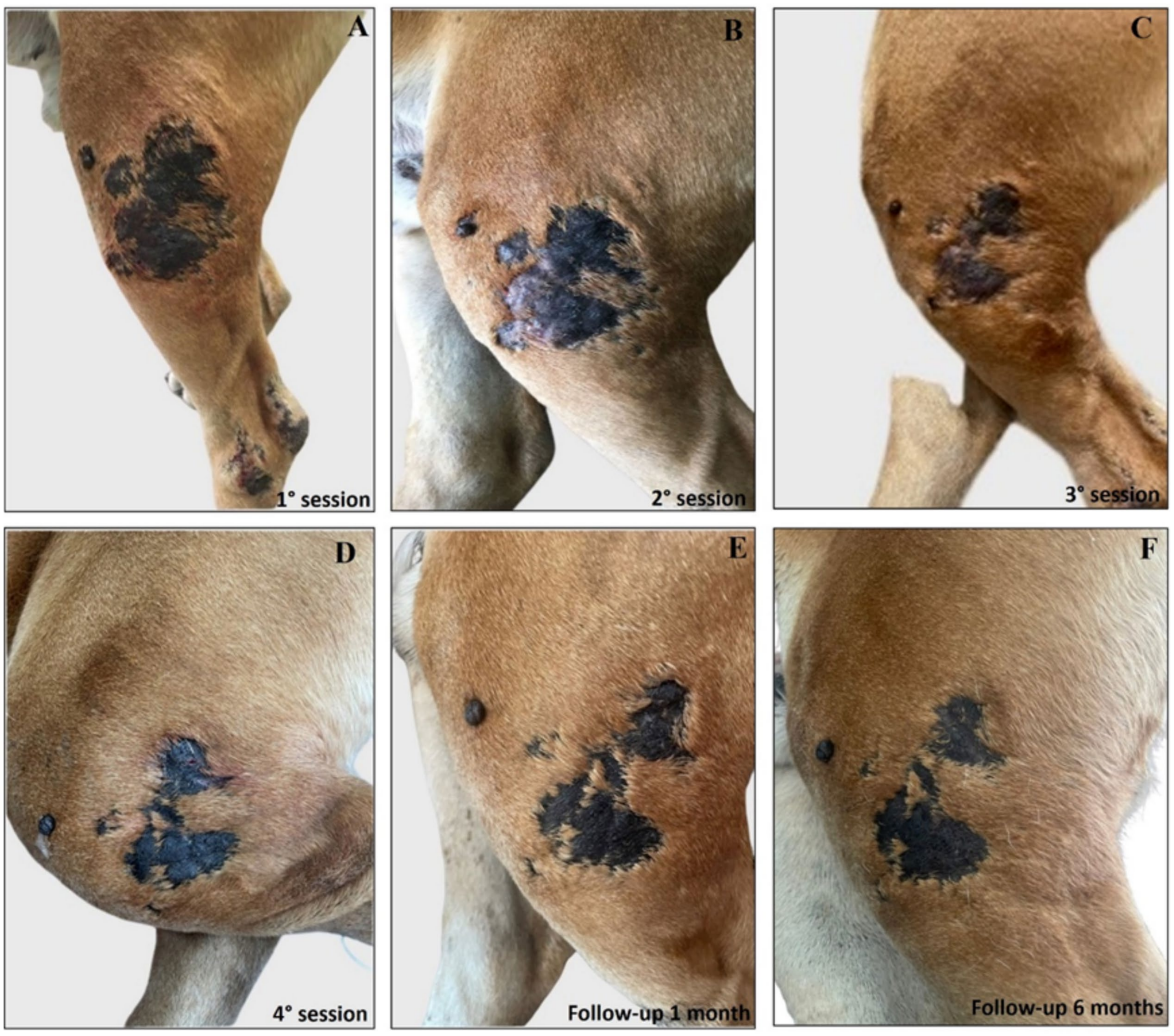
Treatment evolution of left hindlimb femoral lesions with Glucantime®. Representative images of femoral lesions on the left hindlimb during the Glucantime® experimental protocol. The images show femoral lesions before the beginning of the subcutaneous injection of Glucantime® **(A)**, before every subsequent session **(B–D)**, and at 1 month **(E)**, and 6 months of follow-up **(F)**.

**Figure 8:**
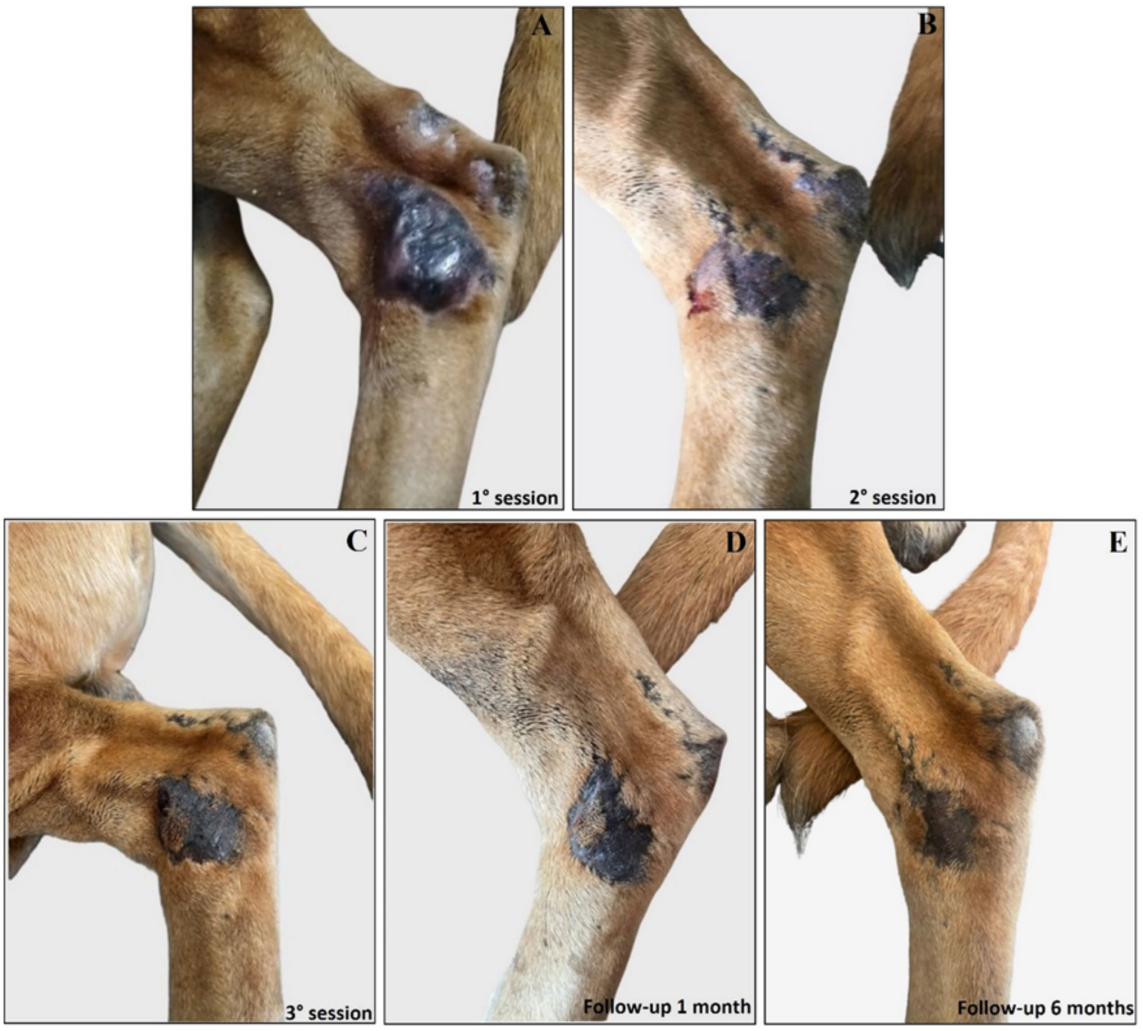
Treatment evolution of left hindlimb tibio/tarsal lesions with Glucantime®. Representative images of tibio/tarsal lesions of the left hindlimb during the Glucantime® experimental protocol. The images show tibio/tarsal lesions before the beginning of subcutaneous injection of Glucantime® **(A)**, before every subsequent session **(B,C)**, and at 1 month **(D)** and 6 months of follow-up **(E)**.

Interestingly, the experimental localized treatment with Glucantime® has proven effective in counteracting skin lesions caused by *Leishmania* infection, leading to a progressive improvement after each treatment session of the right hindlimb femoral lesion (Figures 6A–E), left hindlimb femoral lesion (Figures 7A–D), and of the left tibio/tarsal lesion (Figures 8A–C).

Complete healing was observed 1 month after the end of the treatment cycle (Figures 6F, 7E, 8D) and maintained after 6 months (Figures 6G, 7F, 8E), when the lesions appeared smaller, drier, and no longer itchy and purulent. During the entire treatment and monitoring period, the dog was in good general physical condition. Furthermore, no gammopathy [serum *γ*-globulin concentration, 10.4% (reference range, 5 to 15%)] or alterations in the serum hematologic and biochemical profile were detected at 6-month follow-up (Supplementary material 3).

## Discussion

Leishmaniasis is endemic in tropical, subtropical, and temperate regions in 98 countries, including all southern countries of Europe (12, 68). In these regions, *Leishmania infantum* is identified as the causative agent of the disease, with an average of approximately 700 autochthonous human cases annually reported (13, 68). In Europe, *Leishmania infantum* is the causative agent of canine leishmaniasis, and dogs are the main reservoir hosts of the zoonotic type of both VL and CL, exhibiting a seroprevalence of up to 30% (12, 68).

In this study, we have described the case of a dog residing in Calabria, a region of southern Italy. Following a diagnosis of leishmaniasis, the dog, physically asymptomatic, underwent the standard therapeutic protocol based on miltefosine and allopurinol.

Miltefosine has been increasingly used by veterinarians over the last 15 years, with some undeniable advantages over the use of pentavalent antimonials, such as oral administration and the consequent reduction of mild adverse effects (42). Furthermore, having the canine treatment available was of great importance, since, as reported in several cases, the therapy provides clinical improvement and a reduction of the animal’s infectivity toward sandflies (50).

However, therapeutic failures were reported both in monotherapy and in combination therapy with other drugs, such as allopurinol (54). Although the symptoms are reduced, the parasites are not always eradicated, and relapse can occur (55, 56). Several cases of induced resistance and animals remaining susceptible to infection by the invertebrate vector despite clinical recovery were reported (58).

In the present clinical case, at the end of the treatment cycle, the gammopathy previously observed regressed; however, the dog was referred due to the appearance of suspicious non-healing cutaneous lesions.

In our opinion, this reoccurrence was probably due to the partial efficacy of parenteral therapy, which is significantly reduced when *Leishmania* lesions appear in peripheral and superficial areas, as in this clinical case.

It is feasible that the active ingredient administered orally at the recommended dose is unable to reach effective concentrations in peripheral areas, such as the skin of the limbs, where capillary circulation does not allow sufficient drug supply and absorption. Preclinical studies indicated a broad distribution and absorption of miltefosine predominantly in the kidney, adrenal glands, intestinal mucosa, liver, and spleen (69, 70). Furthermore, the absence of signs of generalized disease led to the assumption that localized cutaneous leishmaniasis could represent the site of the parasite inoculation (71, 72). Therefore, localized pharmacological treatment could be a valid alternative.

In humans, intralesional administration of pentavalent antimonial was used since 1980s to treat CL in the Old World (61–63). In 2010, the World Health Organization (WHO) recommended the use of safer and less toxic lesion-targeted treatments (65, 66), especially in patients with contraindications to the systemic use (64), for the control of leishmaniasis.

Recent studies conducted on patients with CL have compared systemic administration of meglumine antimoniate with intralesional ones, suggesting that localized treatment of CL lesions is a simple, efficient, and safe option (63, 67). Furthermore, treatment of single and/or small lesions by subcutaneous administration of meglumine antimoniate reduces the risk of dissemination to the mucosa by directly eliminating the parasites (71, 72). Although published scientific evidence on localized treatments of cutaneous leishmaniasis in dogs using meglumine antimoniate is limited, and small numbers of animals were used in most of the available studies, existing data report encouraging results. More than 20 years ago, Barbosa Santos et al. already recommended intralesional therapy as the first choice in the treatment of canine integumentary leishmaniasis due to its efficacy. In their study, 25 adult dogs, naturally infected with *L. braziliensis* showing ulcerated skin lesions, mucosal lesions, or multiple lesions, were treated with N-methylglucamine antimonate at a dose of 85 mg Sbv + by the intralesional route. The dogs were observed for 6 months after the last dose, and serial evaluation of antibodies by Indirect Fluorescence Antibody Test (IFAT) demonstrated that 16% of the treated dogs showed a decrease in titer, 21% tested seronegative, and 86.6% experienced completely healed lesions (73).

In a recent randomized control study, 32 domestic dogs, which received the recommended vaccines for dogs in Brazil and were in good nutritional status, showed cutaneous or muzzle lesions that were treated with 5 mL of intralesional Glucantime® on days 0, 15, and 30. Interestingly, on day 90, the healing rate was 87.5% in the tested group compared to those who received 12.5% 0.9 NaCl (74).

The existing knowledge and protocols regarding intralesional therapy with meglumine antimoniate in human patients with CL as well as the positive outcomes observed in the studies discussed above on leishmanial dogs support the findings observed in our clinical case.

In our case report, the occurrence of infection in the non-healing tibio/tarsal lesion, confirmed by cytology, was initially treated with amoxicillin+clavulanic acid, metronidazole, prednisone and treatment with allopurinol was resumed; however, after 6 months, the owner reported a substantial clinical decline in the condition of the dog, with worsening and spreading of the lesions. These findings were also associated with the identification of renal microlithiasis and mild splenopathy using ultrasound, outlining a possible clinical presentation of a leishmaniotic subject.

Finally, complete healing of the cutaneous lesions was achieved only through subsequent experimental treatment of Glucantime® through peripheral subcutaneous injections performed once a month for 5 months and subsequent follow-up at 1 and 6 months after the completion of the treatment cycle.

Throughout the treatment and monitoring period, the dog remained in good overall physical condition, demonstrating complete healing of the lesions as early as 1 month after the end of the cycle and maintained for another 6 months.

This clinical case highlights how localized treatment of lesions has proven to be more effective and less toxic in managing the infection than prolonged systemic treatment, which remains the first choice in the treatment of canine leishmaniasis.

## Conclusion and perspective

In conclusion, the present clinical case highlights how experimental localized subcutaneous injections of meglumine antimoniate (Glucantime®) on Boxer *Leishmania infantum* skin lesions has proven to be more effective and less toxic in managing the infection than prolonged systemic treatment, which, however, remains the first choice in the treatment of canine leishmaniasis.

In this perspective, further pre-clinical and clinical randomized and controlled studies on the pharmacokinetics and bioavailability of existing and novel leishmanicides will be conducted to determine optimal dosing regimens, administration route strategies, and combination therapies that may play a key role in the successful targeted treatment of canine leishmaniasis.

In addition, the implementation of leishmaniasis prevention programs based on screening, vaccination, and use of topical insect repellents (60) should be complemented by a thorough review of current pharmacological tools to define targeted therapeutic protocols useful for improving efficacy, reducing toxicity, and limiting the appearance of resistance.

## Data Availability

The original contributions presented in the study are included in the article/Supplementary material, further inquiries can be directed to the corresponding authors.
