# Supplementary material for: Efficacy of meglumine antimoniate treatment on boxer Leishmania infantum skin lesions: case report
Source: Front Vet Sci. 2025 Jun 30;12:1600004. doi: 10.3389/fvets.2025.1600004 (PMC12258295; doi:10.3389/fvets.2025.1600004)
Supplement: Supplementary file 1 [file Data_Sheet_1.pdf]

Io sottoscritto ________________________________ GENNARO CARRESI_______________________________________
(cognome e nome)

Indirizzo:VIA EMILIO CUZZOCREA N. 15, Cap: 89127, Comune: REGGIO CALABRIA____________Provincia____RC__

Proprietario/a o Affidatario/a di:_____________________ETTORE___MICROCHIP N.380260000905119___________
(nome e microchip dell'animale)

- [X] CANE
- [ ] GATTO
- [ ] ALTRO

Razza:______BOXER_________________________Sesso:______M___________:____________età:_____7 ANNI_____________

AUTORIZZO

al trattamento dei dati clinici del cane sopra indicato a fini scientifici di studio e pubblicazione.

Data:___25/09/2023_________________

Firma

____
(il proprietario o l'affidatario dell'animale)
