# Supplementary material for: Efficacy of meglumine antimoniate treatment on boxer Leishmania infantum skin lesions: case report
Source: Front Vet Sci. 2025 Jun 30;12:1600004. doi: 10.3389/fvets.2025.1600004 (PMC12258295; doi:10.3389/fvets.2025.1600004)
Supplement: Supplementary file 3 [file Data_Sheet_3.pdf]

MODULO DI CONSENSO INFORMATO

Clinica Veterinaria Tripodi, Via Glauco, 6, 89123 Reggio Calabria RC

Io sottoscritto ________________________________ GENNARO CARRESI_______________________________________
(cognome e nome)

Indirizzo:VIA EMILIO CUZZOCREA N. 15, Cap: 89127, Comune: REGGIO CALABRIA____________Provincia____RC__

Proprietario/a o Affidatario/a di:_____________________ETTORE___MICROCHIP N.380260000905119___________
(nome e microchip dell'animale)

- [X] CANE
- [ ] GATTO
- [ ] ALTRO

Razza:______BOXER_________________________Sesso:______M___________:___________età:_____7 ANNI____________

DICHIARO di essere consapevole che l'animale sopraindicato, debba essere sottoposto:

ad una somministrazione intra lesionale di Glucantime®, da ripetere ogni trenta giorni.____

DICHIARO di essere stato informato in modo chiaro e per me comprensibile, con particolare riguardo a quanto sopra, sia dei benefici che dei rischi generici e specifici e le possibili complicanze che ne possano derivare anche qualora il trattamento sanitario concordato venga espletato secondo tutte le tecniche dell'arte, con perizia e diligenza e di accettarli integralmente.

DICHIARO di avere ricevuto in merito all'animale sopraindicato un'informazione comprensibile, adeguata ed esauriente:

- sulla modalità di effettuazione del trattamento e sulla via di somministrazione proposta;
- sui vantaggi, il grado di efficacia nonché sulle possibili conseguenze sanitarie derivanti dal trattamento;
- sugli eventuali effetti collaterali e probabilità del loro verificarsi, nonché sulle modalità di trattamento;
- sulla possibilità di richiedere, in qualsiasi momento, un ulteriore colloquio, per poter acquisire maggiori informazioni;
- sulla possibilità di revocare il presente consenso in qualsiasi momento, con conseguente mancata o ridotta somministrazione del trattamento.

DICHIARO, inoltre, di essere a conoscenza che le condizioni generali di salute del mio animale sono così descrivibili:
il cane di età 7 anni si presenta in buono stato di salute generale, compatibile con l'età, ma da mesi presenta delle lesioni nodulari infiammatorie e purulente, poco responsive ai trattamenti antibiotici e antinfiammatori, sia locali che sistemici, localizzate agli arti posteriori a livello del tarso e del ginocchio.

ESSENDOMI STATO PROSPETTATO quanto sopra,
HO DECISO di:

- [X] Accettare il trattamento sanitario propostomi: esami emato-biochimici, ecografia addome e cardio, somministrazione off-label di Glucantime® all'interno delle lesioni cutanee presenti sugli arti posteriori.
- [ ] non accettare il trattamento sanitario propostomi, sotto la mia piena responsabilità;
- [ ] di optare, sotto la mia piena responsabilità, per un profilo meno esteso come segue

Pertanto, nella mia piena capacità di intendere e di volere e preso atto della situazione illustratami,

AUTORIZZO

la Dott.ssa Ferrucci Clara Francesca, medico veterinario, ad effettuare sull'animale sopra indicato le procedure **diagnostiche e terapeutiche** necessarie che mi sono state prospettate.

Inoltre, dichiaro, di essere stato informato che lo stato di salute dell'animale di cui sopra, dipende anche dalla diligente osservazione da parte mia delle prescrizioni e delle indicazioni medico-veterinarie fornitemi.

Data:___25/09/2023_________________

Firma

____

(il proprietario o l'affidatario dell'animale)
