# Supplementary material for: Efficacy of meglumine antimoniate treatment on boxer Leishmania infantum skin lesions: case report
Source: Front Vet Sci. 2025 Jun 30;12:1600004. doi: 10.3389/fvets.2025.1600004 (PMC12258295; doi:10.3389/fvets.2025.1600004)
Supplement: Supplementary file 4 [file Image_1.pdf]

Supplementary Figures, Materials and Legends

Supplementary Figure 1. Serum protein electrophoresis report at the end of miltefosine treatment.

BieffeLAB Analisi veterinarie Dr Bruno Crucitti P.Iva 02735620805

bieffeLAB
laboratorio di analisi

Reggio Cal 01/03/22

Proprietario: Carresi Nome: ettore
Specie: Cane Razza: boxer
Sesso: M Età: 9aa
Med. Vet.: Dott.ssa Clara Ferrucci

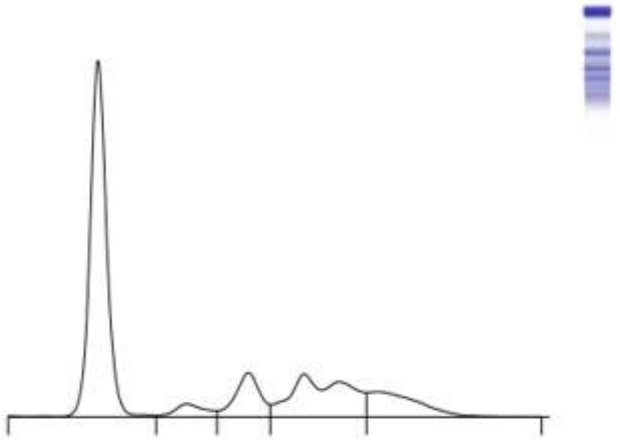

ELETTROFORESI DELLE SIEROPROTEINE

Frazioni		%	Int. Rif.
Prot. Tot.	7.4 g/dl		
Rapp. A/G		1.19	(0.65 – 1.3)
Albumina		54.3	(50.0 – 65.0)
Alfa1		3.6	(2.0 – 6.0)
**Alfa2**		**9.7**	**(10.0 – 18.5)**
Beta		21.1	(14.0 – 22.0)
Gamma		11.3	(6.0 – 15.0)

Il Direttore Sanitario
Bruno Crucitti Med Vet

Via loreto,99 Reggio Calabria 89133 Tel **0965 594080** E-mail info@bieffelab.it

Supplementary Figure 2

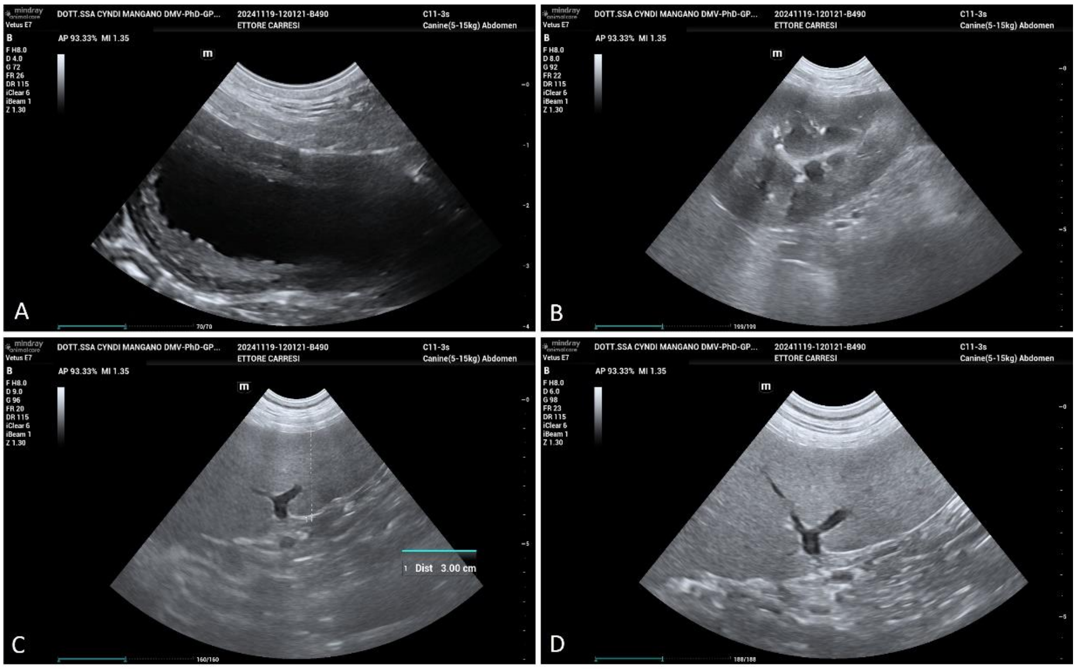

Supplementary Figure 2. Representative abdominal ultrasound images performed after the end of antibiotic therapy. The analysis performed with a Mindray Vetus E7 ultrasound system showed bladder jagged with lithiasis (**A**), left kidney characterized by hyper-echogenicity of the renal pillars compatible with renal lithiasis(**B**) and spleen increased in volume and with non-homogeneous parenchyma (**C** and **D**).

Supplementary Material 1. Report of abdominal ultrasound performed after the end of antibiotic therapy.

Supplementary Material 2. Echocardiographic report performed after the end of antibiotic therapy.

Supplementary Material 3. Serum hematologic and biochemical analyses and serum protein electrophoresis report at 6-month follow-up after the end of experimental intralesional treatment with Glucantime®.
