# Supplementary material for: Efficacy of meglumine antimoniate treatment on boxer Leishmania infantum skin lesions: case report
Source: Front Vet Sci. 2025 Jun 30;12:1600004. doi: 10.3389/fvets.2025.1600004 (PMC12258295; doi:10.3389/fvets.2025.1600004)
Supplement: Supplementary file 5 [file Supplementary_file_1.pdf]

DOTT.SSA CYNDI MANGANO DMV-PhD-GPCERT DI

Nome:ETTORE CARRESI
ID animal:20230608-103727-B490
Età:10Anni
Sex: Masc

Telephone:3492183280
Fax:3492183280
SitoWeb:cyndi_m@hotmail.it

Canine(>15kg) Abdomen
Data esam:08/06/2023
Medico rif.:TRIPODI
Attrezz usata: Mindray Vetus E7
Operatore:DOTT.SSA CYNDI MANGANO

Indicazioni e dati clinici
Weight:38.00kg **BSA:**1.14m²

Addome - 1/5 Page

Immag ultrasuoni

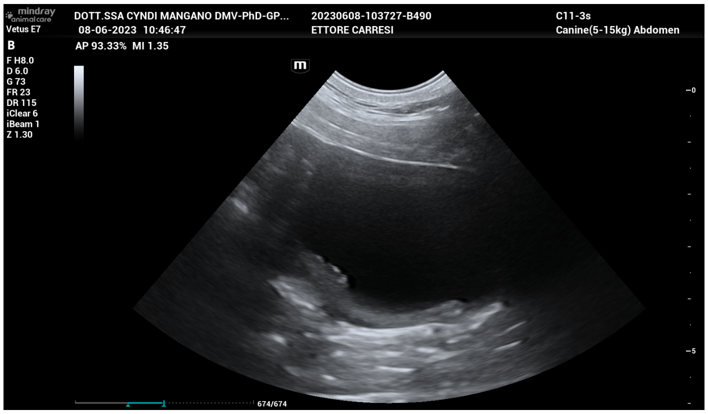

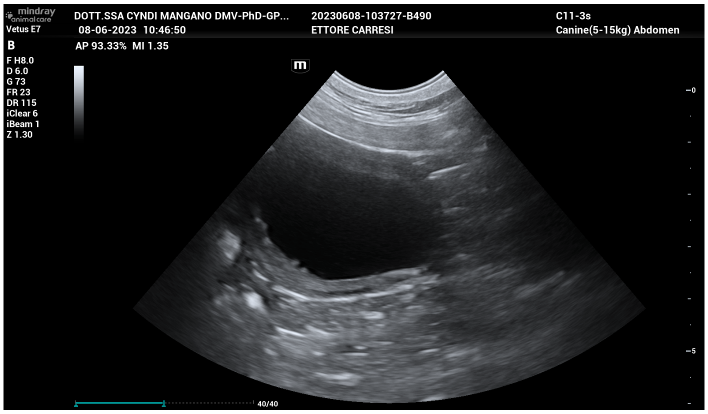

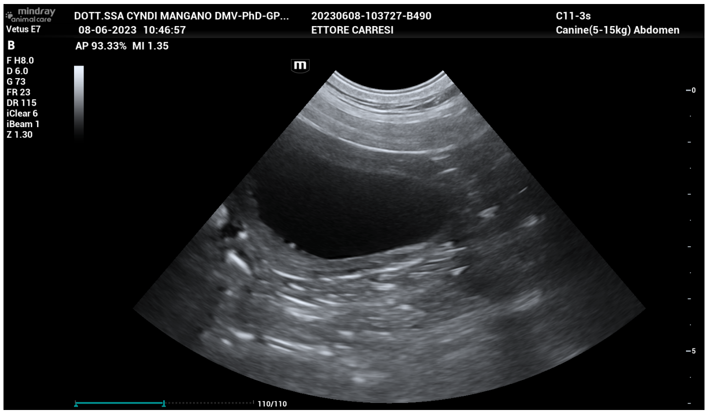

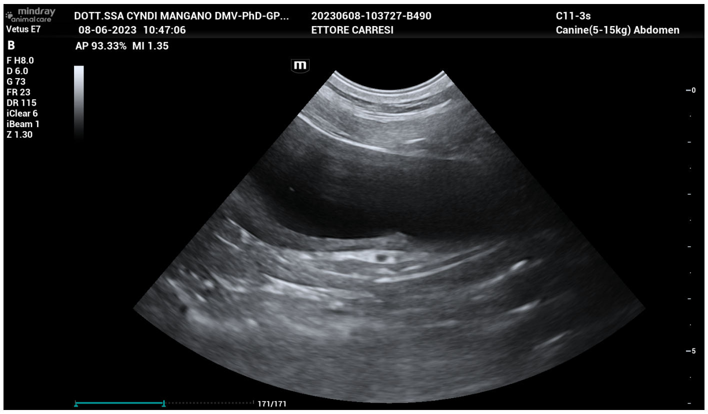

Nome:ETTORE CARRESI
ID animal:20230608-103727-B490 **Età:**10Anni **Sex:**Masc

Canine(>15kg) Abdomen

Data esam:08/06/2023 **Medico rif.:**TRIPODI

Addome - 2/5 Page

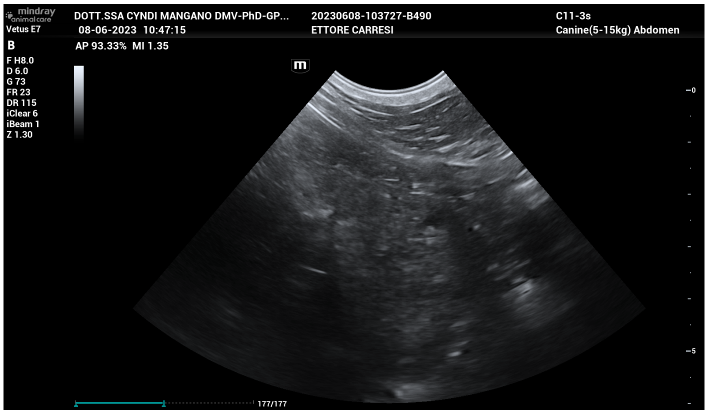

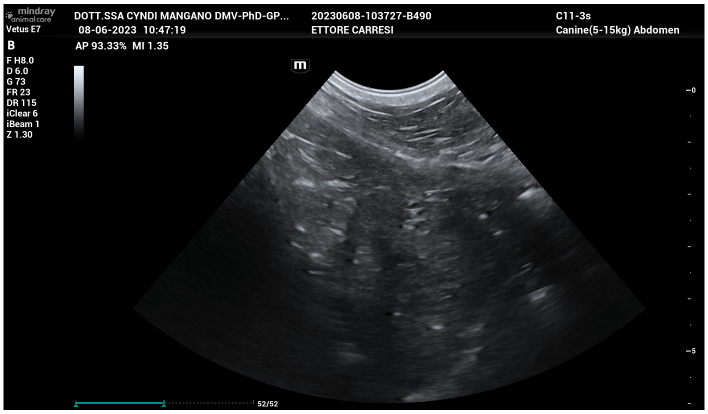

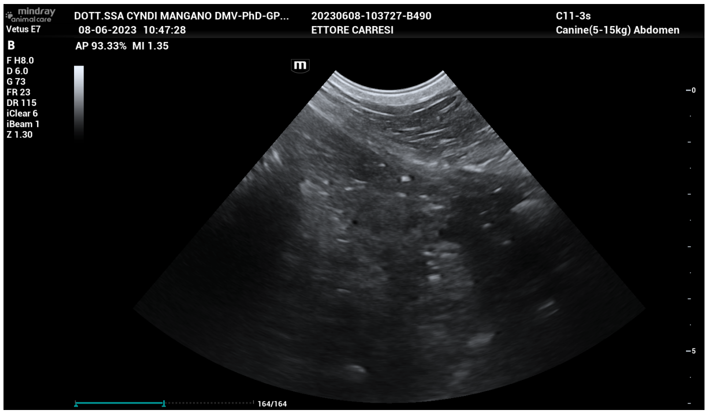

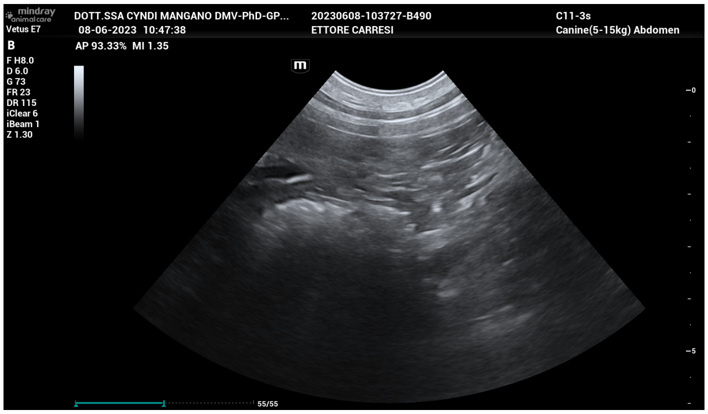

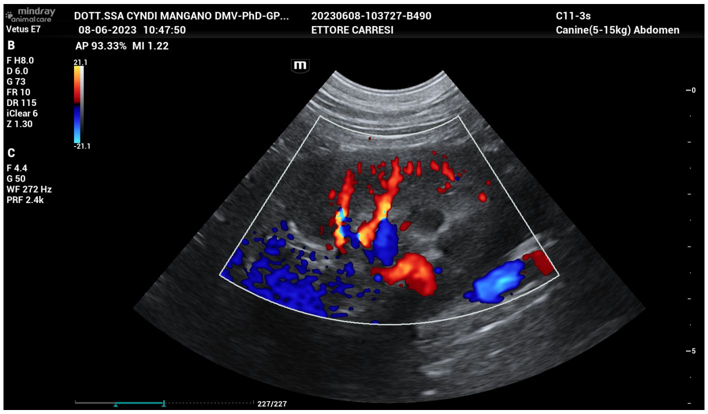

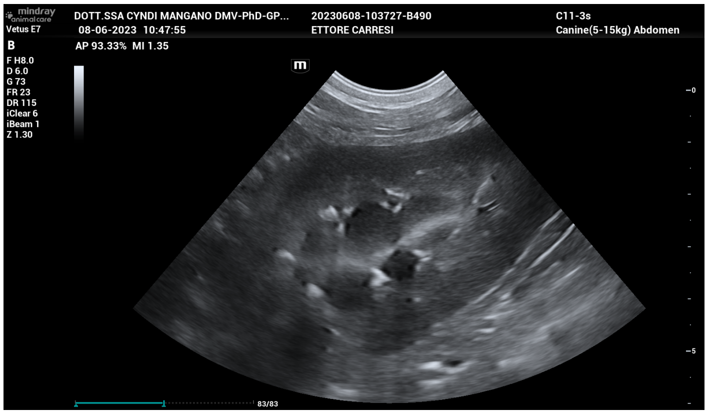

Nome:ETTORE CARRESI
ID animal:20230608-103727-B490 **Età:**10Anni **Sex:**Masc

Canine(>15kg) Abdomen

Data esam:08/06/2023 **Medico rif.:**TRIPODI

Addome - 3/5 Page

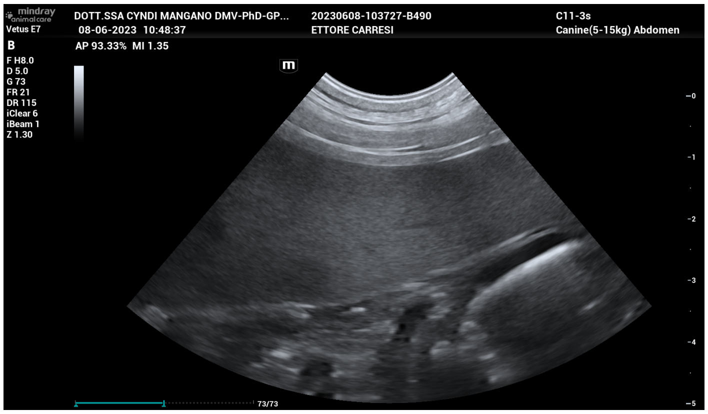

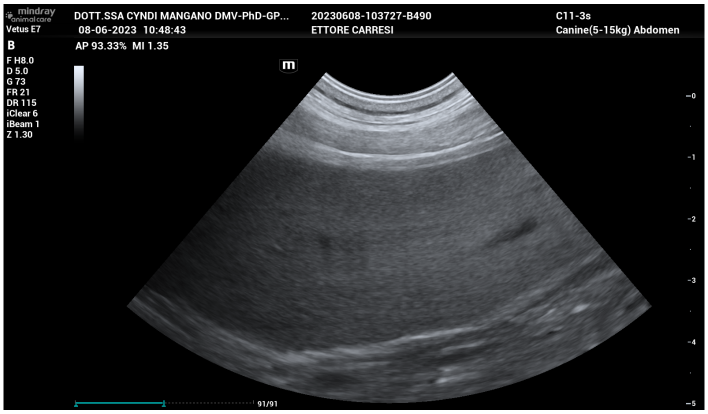

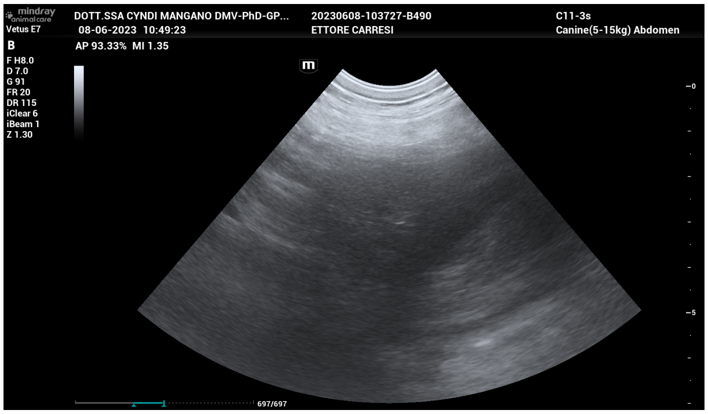

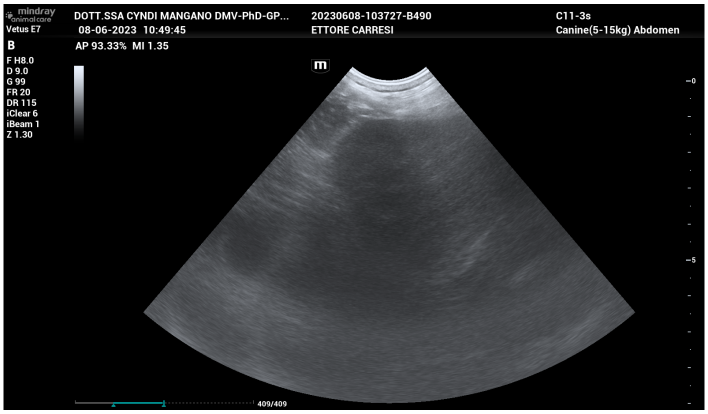

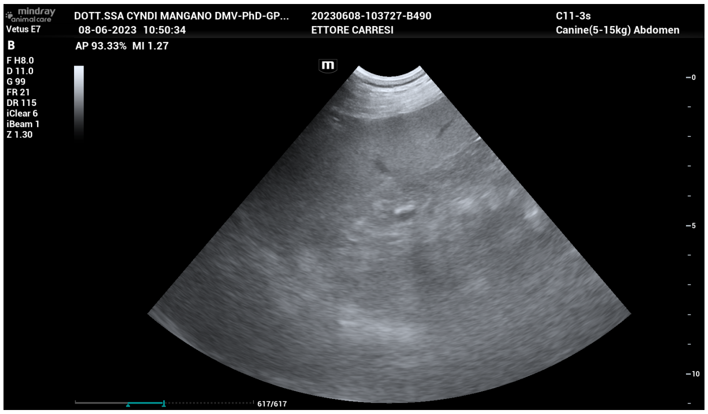

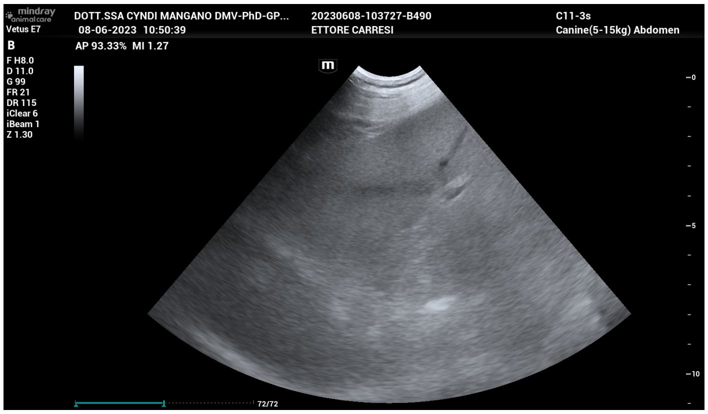

Nome:ETTORE CARRESI
ID animal:20230608-103727-B490 **Età:**10Anni **Sex:**Masc

Canine(>15kg) Abdomen

Data esam:08/06/2023 **Medico rif.:**TRIPODI

Addome - 4/5 Page

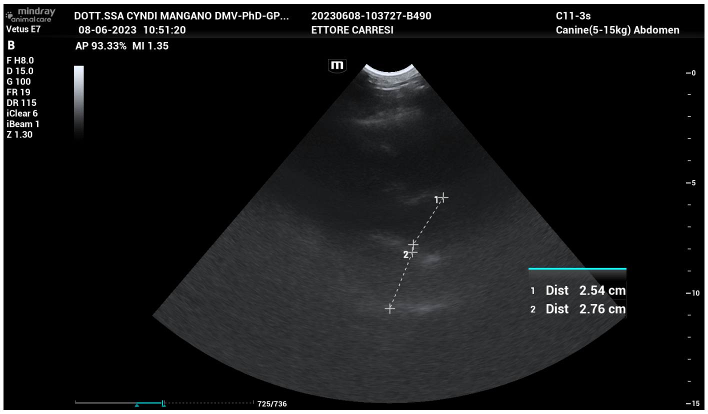

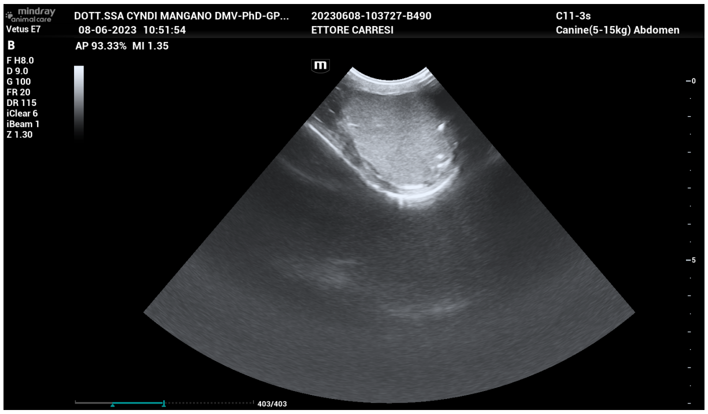

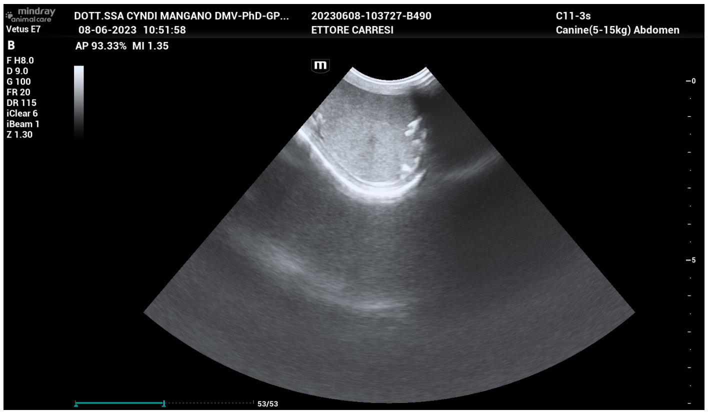

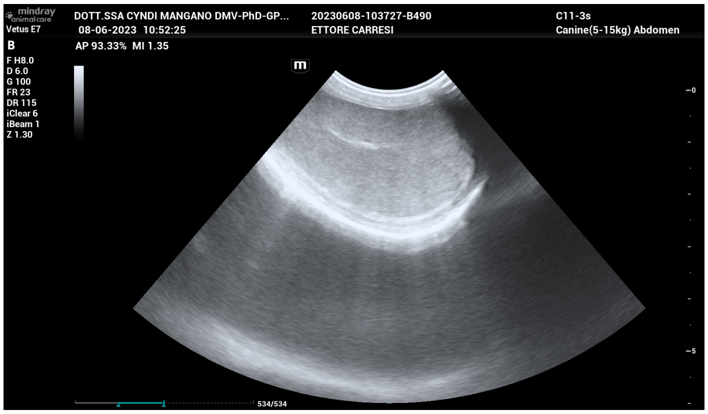

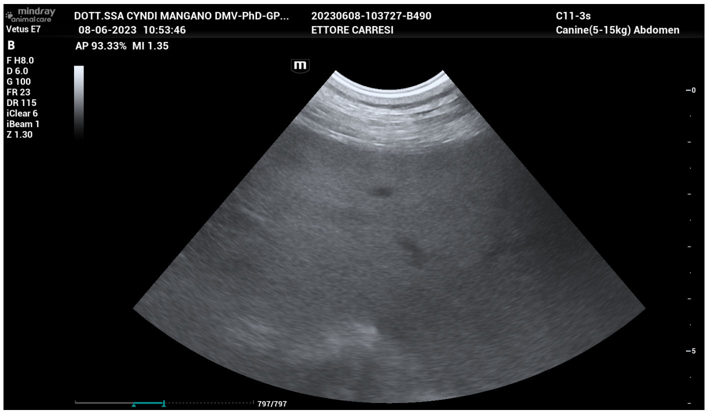

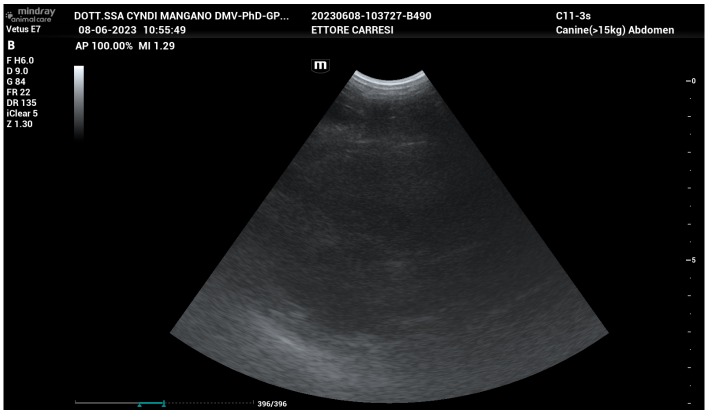

Nome:ETTORE CARRESI
ID animal:20230608-103727-B490 **Età:**10Anni **Sex:**Masc

Canine(>15kg) Abdomen

Data esam:08/06/2023 **Medico rif.:**TRIPODI

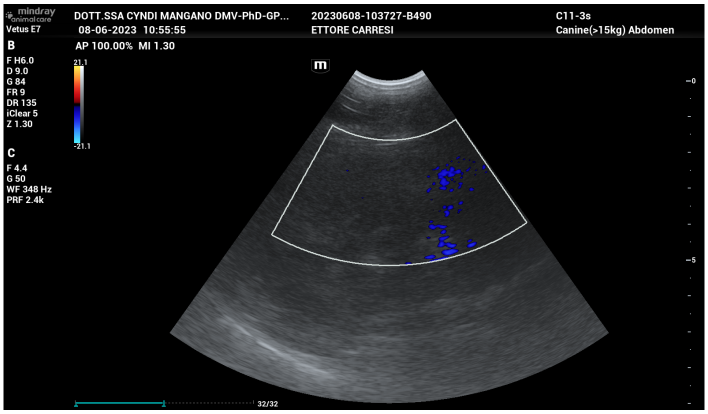

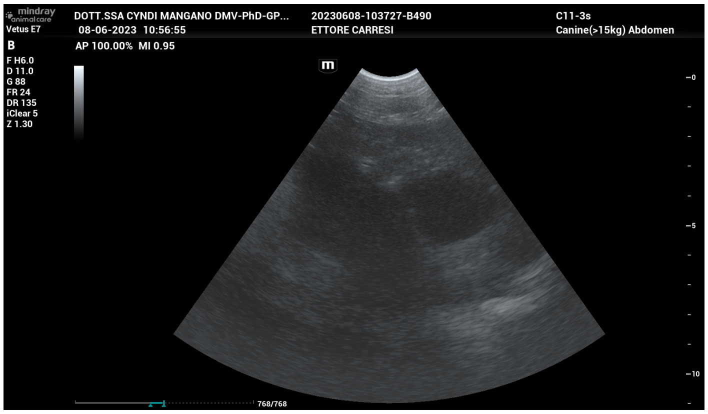

Comm.:

CANE BOXER MASCHIO INTERO 10A 38KG
ESAME DI CONTROLLO ADDOME
PAZIENTE COLLABORATIVO
Findings:
VESCICA > in sede - normocapace - parete normale - contenuto anecogeno
RENI SX E DX > in sede - forma dimensioni normali - rapporto cortico-midollare mantenuto - pelvi normale - ecogenicita' normale - vascolarizzazione normale - microlitiasi a livello dei pilastri
SURRENALI SX E DX > in sede - forma dimensioni normali - ecogenicita' normale - vascolarizzazione normale
MILZA > in sede - forma volume aum- ecogenicita' aum- parenchima con aree ipoecogene ma a margini diffusi- vascolarizzazione normale - assenza di lesioni focali
FEGATO > in sede - forma volume normali - ecogenicita' normale - parenchima omogeneo - vascolarizzazione normale - assenza di lesioni focali - dotti nella norma
CISTIFELLEA > in sede - normocapace - parete normale - contenuto anecogeno
PANCREAS >forma volume normali - ecogenicita' normale - parenchima omogeneo - vascolarizzazione normale - assenza di lesioni focali
STOMACO > in sede - stratificazione e spessore di parete normale - contenuto normale
INTESTINO > stratificazione e spessore di parete normale - contenuto normale - peristalsi presente e normale
COLON > stratificazione e spessore di parete normale - contenuto fecale normale
RIPRODUTTORE > microcisti prostatiche - microlitiasi testicolare
CUORE> rapp atrio ao nella norma
Diagnosis:
microlitiasi renale
spleopatia moderata
ipb + microlitiasi testicolare
Comments:
quadro da leish+

Firma (sigillo): **Data firma:**
