# Supplementary material for: Efficacy of meglumine antimoniate treatment on boxer Leishmania infantum skin lesions: case report
Source: Front Vet Sci. 2025 Jun 30;12:1600004. doi: 10.3389/fvets.2025.1600004 (PMC12258295; doi:10.3389/fvets.2025.1600004)
Supplement: Supplementary file 6 [file Supplementary_file_2.pdf]

DOTT.SSA CYNDI MANGANO
DMV-PhD-GPCERT DI

Telephone:3492183280
Fax:
SitoWeb:

Nome:ETTORE **Animale:**Cane (>15 kg)
Osp.:CARRESI
ID animal:20221004-173207-B490
Età:9Anni
Sex: Masc **Sterilizzazione:**No

Canine(>15kg) Cardiac

Data esam:04/10/2022
Medico rif.:TRIPODI
Attrezz usata: Mindray Vetus E7
Operatore:

Indicazioni e dati clinici
Weight:38.00kg **BSA:**1.14m^2

Misure 2D

Aorta & Aortic Valve

LA/Ao(2D)

Diam LA: 3.48cm
Diam Ao: 2.38cm
LA/Ao: 1.47

M Misurazioni

LV

IVSd: 0.70cm
IVSs: 1.26cm
LVIDd: 6.16cm
LVIDs: 4.67cm
LVPWd: 1.44cm
LVPWs: 1.29cm
FS: 24.29%

Teichholz(M)

EDV(Teich): 191.33ml
EDV Index(Teich): 167.58
ESV(Teich): 100.63ml
ESV Index(Teich): 88.14
SV(Teich): 90.69ml
SI(Teich): 79.43
EF(Teich): 47.40%

Immag ultrasuoni

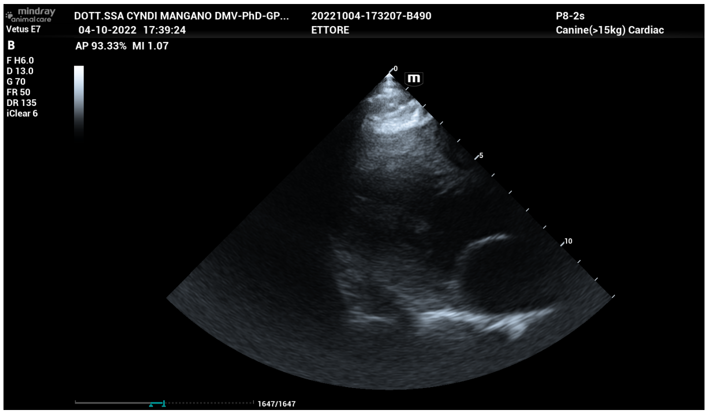

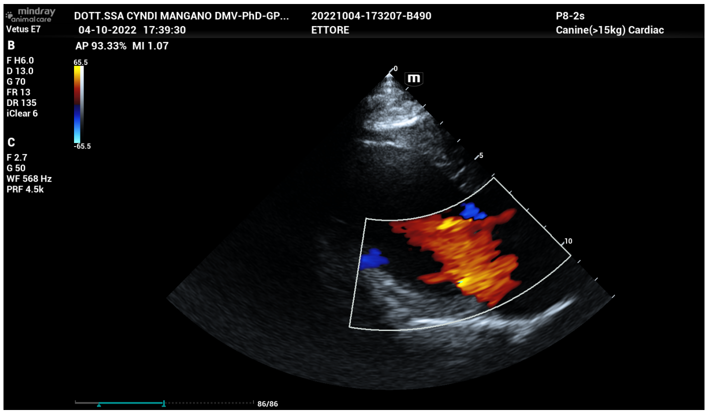

Nome: ETTORE
Osp.: CARRESI
ID animal: 20221004-173207-B490 **Età:** 9Anni **Sex:** Masc

Canine(>15kg) Cardiac

Data esam: 04/10/2022 **Medico rif.:** TRIPODI

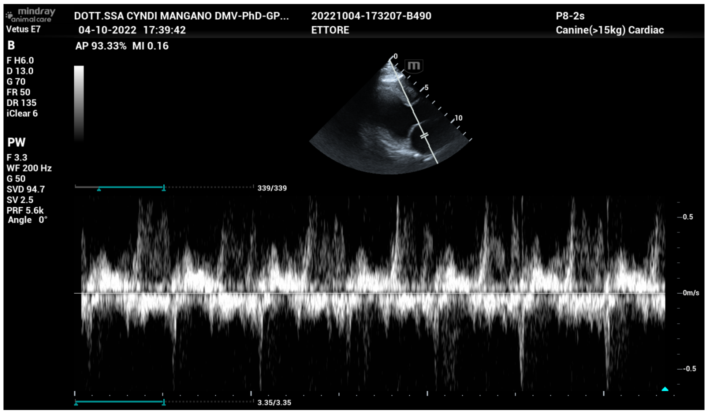

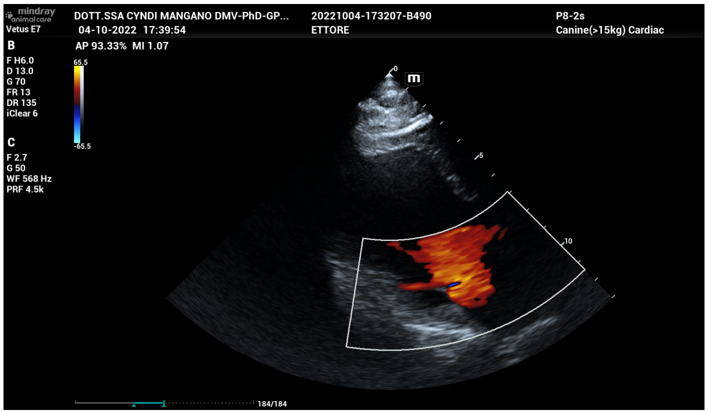

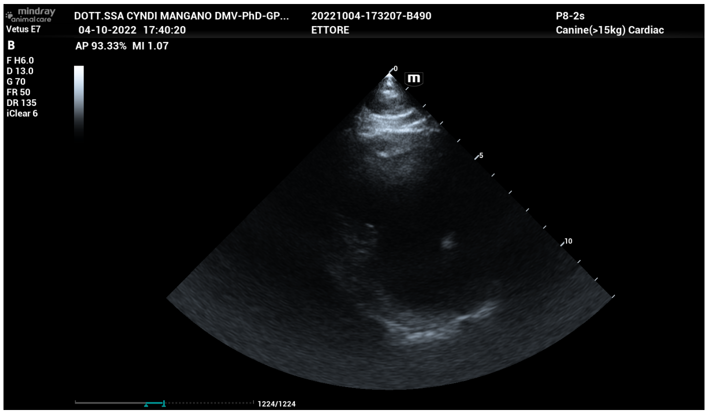

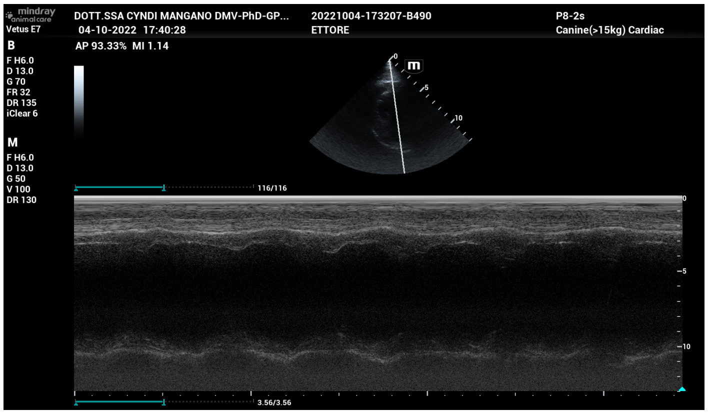

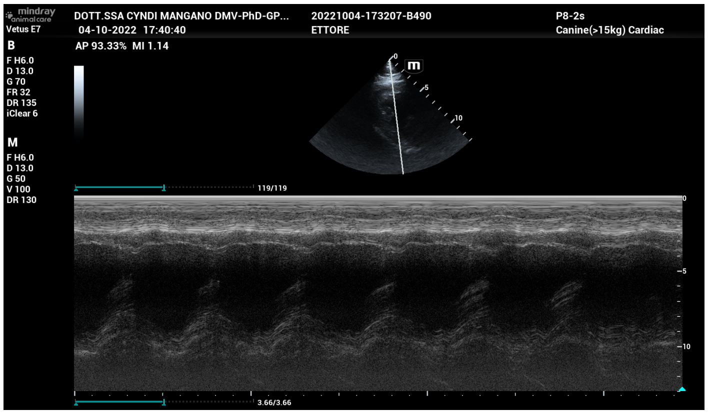

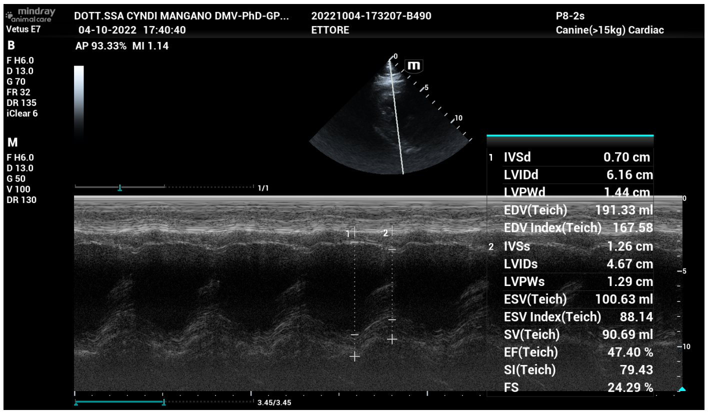

Nome: ETTORE
Osp.: CARRESI
ID animal: 20221004-173207-B490 **Età:** 9Anni **Sex:** Masc

Canine(>15kg) Cardiac

Data esam: 04/10/2022 **Medico rif.:** TRIPODI

Cardiology - 3/5 Page

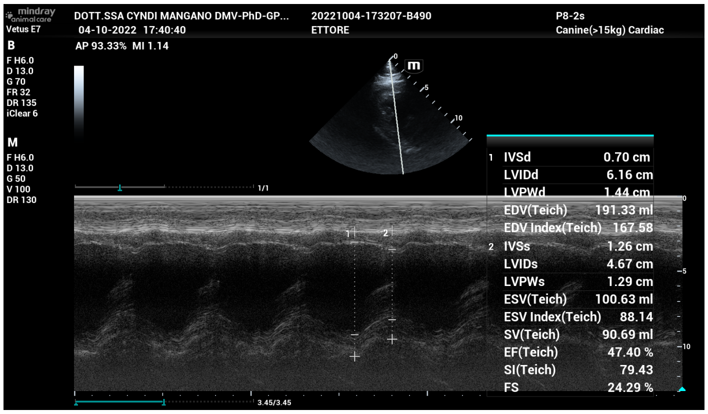

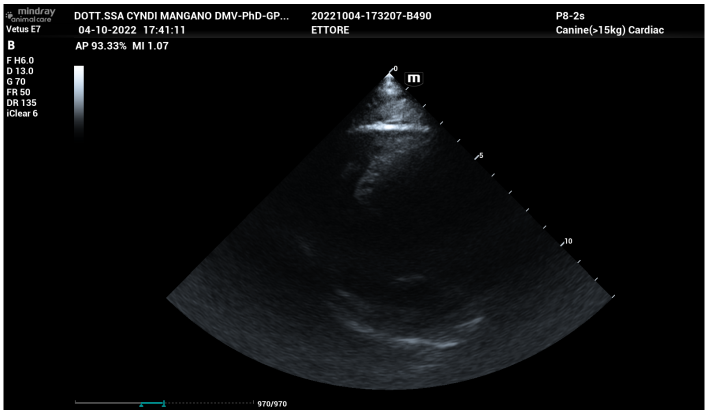

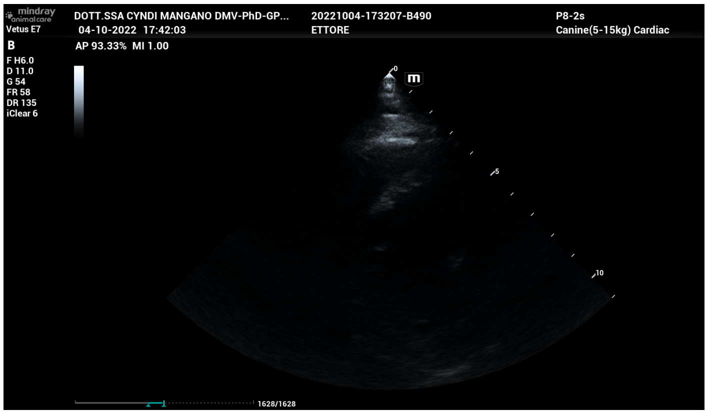

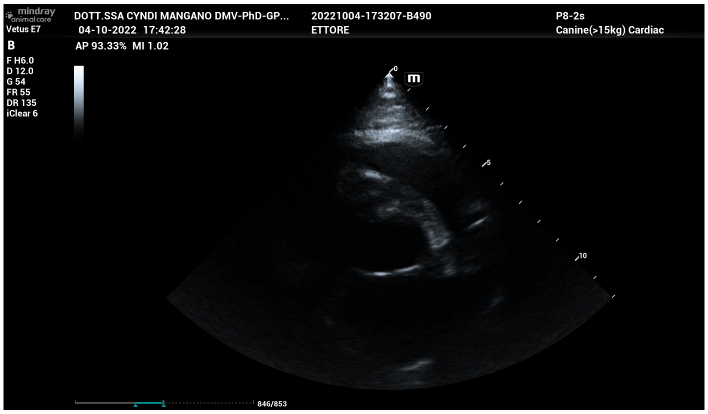

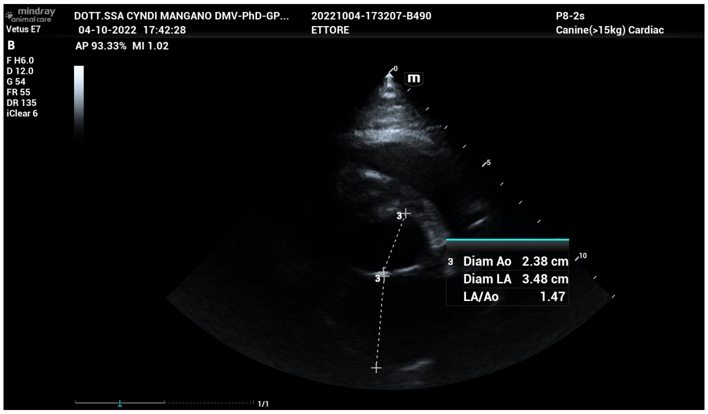

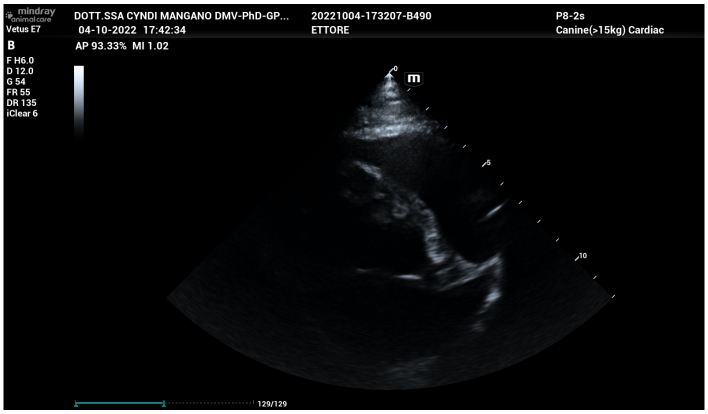

Nome: ETTORE
Osp.: CARRESI
ID animal: 20221004-173207-B490 **Età:** 9Anni **Sex:** Masc

Canine(>15kg) Cardiac

Data esam: 04/10/2022 **Medico rif.:** TRIPODI

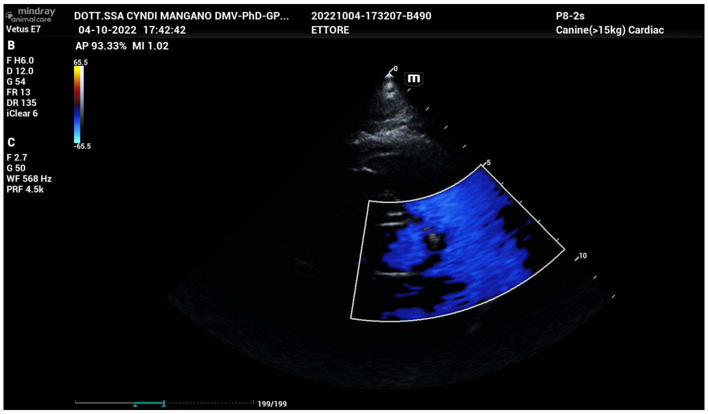

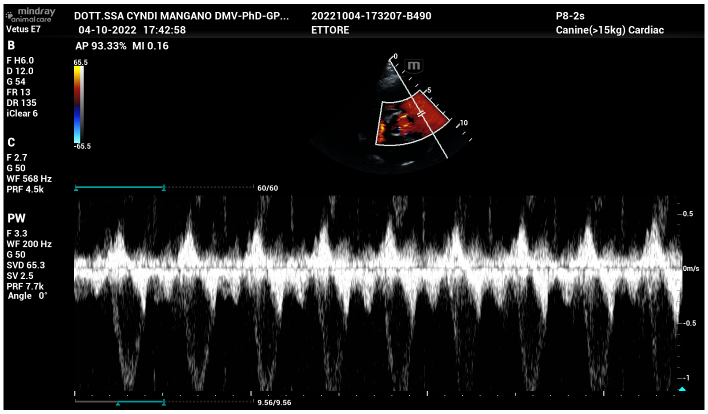

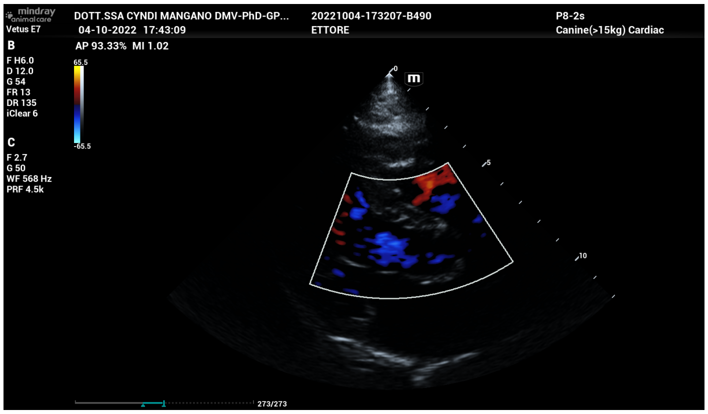

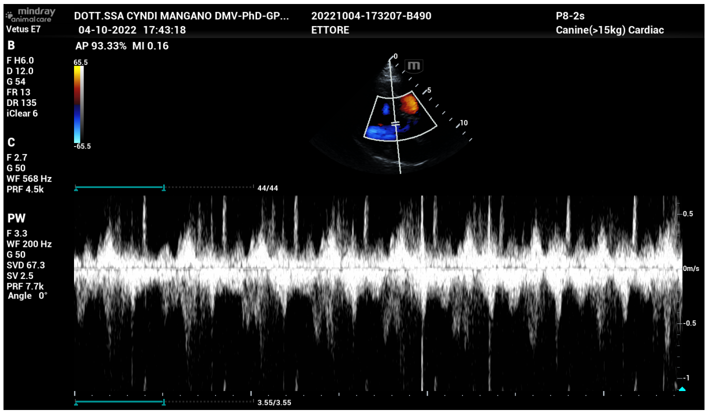

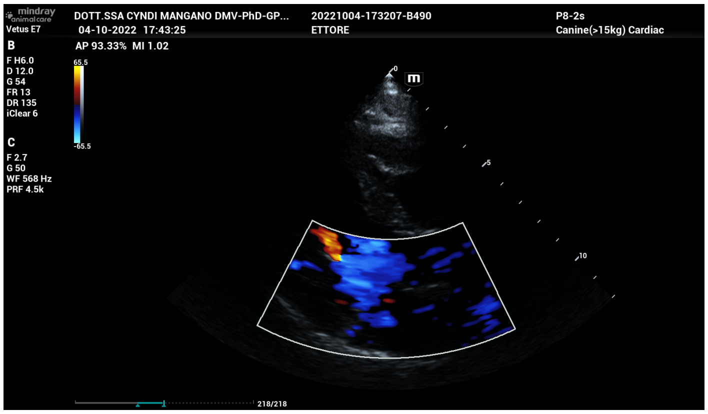

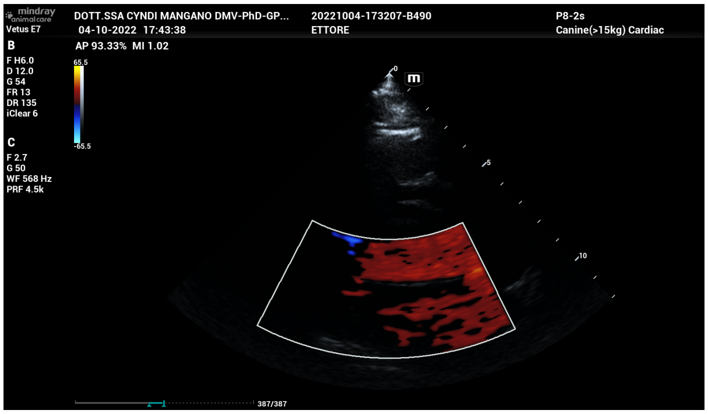

Nome: ETTORE
Osp.: CARRESI
ID animal: 20221004-173207-B490 **Età:** 9Anni **Sex:** Masc

Canine(>15kg) Cardiac

Data esam: 04/10/2022 **Medico rif.:** TRIPODI

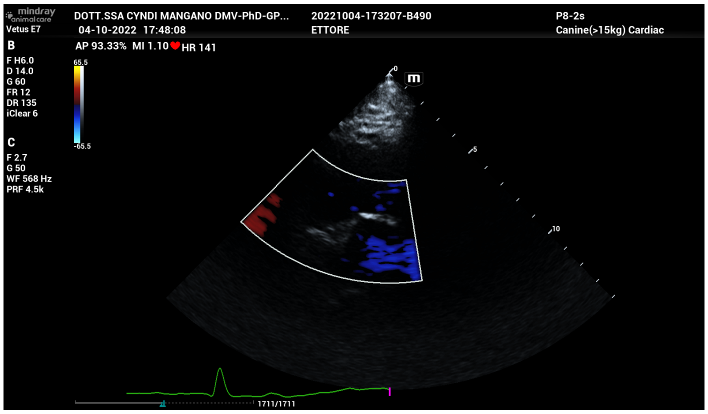

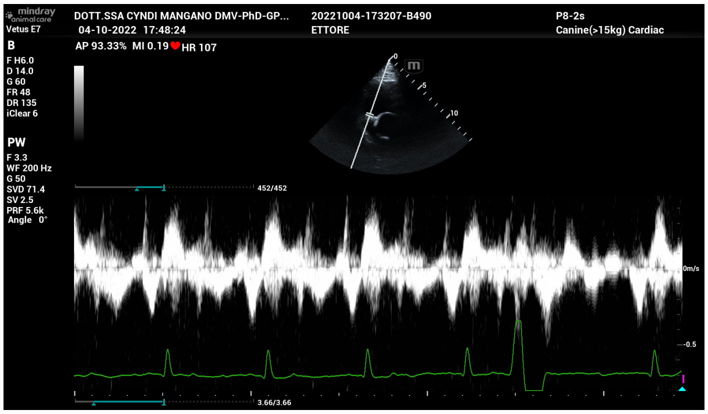

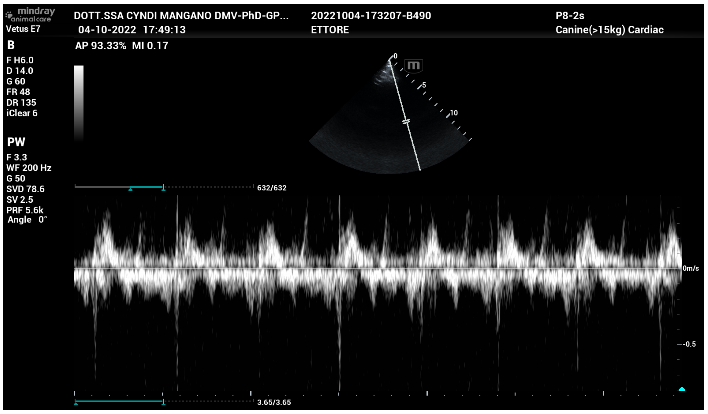

Comm.:

BOXERM INTERO 9A
CONTROLLO AFFANNO - PRECEDENTE ECOCARDIO NORMALE
PAZIENTE COLLABORATIVO
ECG normale ritmo sinusale
COMMENTO assenza di sovraccarico vol e pressorio - mitrale e tricuspide normale - funzionalita diastolica e sistolica mantenuta - aorta e polm normale
fe fs a\ao normale
COMMENTO esame nella norma

Firma (sigillo): **Data firma:**
