# Supplementary material for: Efficacy of meglumine antimoniate treatment on boxer Leishmania infantum skin lesions: case report
Source: Front Vet Sci. 2025 Jun 30;12:1600004. doi: 10.3389/fvets.2025.1600004 (PMC12258295; doi:10.3389/fvets.2025.1600004)
Supplement: Supplementary file 7 [file Supplementary_file_3.pdf]

BieffeLAB Analisi veterinarie Dr Bruno Crucitti P.Iva 02735620805

Reggio Cal 26/09/24

Proprietario: Carresi Nome: ettore
Specie: Cane Razza:
Sesso: Età:
Med. Vet.: Dott.ssa Clara Ferrucci

PROFILO BIOCHIMICO

AST	35.4	10 – 45	UI/L
**ALT**	**76.6**	**10 – 60**	**UI/L**
ALP	32.1	10 – 130	UI/L
GGT	4.4	0.1- 13	UI/L
BIL TOT.	0.15	0.1 – 0.44	mg/dl
PT	7.2	5.8 – 8	g/dl
ALB	2.8	2.6 – 3.8	g/dl
**GLOB**	**4.4**	**2.4 – 4.0**	**g/dl**
**A/G**	**0.6**	**0.7 – 1.5**	
Fe	120	120 – 300	µg/dl
CHOL	176	120 – 300	mg/dl
TRG	31	30 – 95	mg/dl
AMY	1267.3	200 – 1900	UI/L
LIP	197.5	10 – 350	UI/L
UREA	34	15 – 45	mg/dl
CREA	1.1	0.40 – 1.80	mg/dl
Ca	9.5	8 – 12	mg/dl
P	3.5	2.5 – 5.6	mg/dl
GLU	86	70 – 110	mg/dl
LDH	193	30 – 398	UI/L
**CK**	**176.7**	**45 – 155**	**UI/L**
Mg	2.04	1.6 – 2.48	mg/dl
Na	149.4	140 – 155	mEq/L
K	4.32	3.3 – 5.4	mEq/L
Rapp. Na/K	34	> 27	
Cl	117.4	105 – 120	mEq/L

Valori di riferimento validi per la specie canina

NOTE:

Il Direttore Sanitario
Bruno Crucitti Med Vet

Via loreto,99 Reggio Calabria 89133 Tel **0965 594080** E-mail info@bieffelab.it

BieffeLAB Analisi veterinarie Dr Bruno Crucitti P.Iva 02735620805

Reggio Cal 26/09/24

Proprietario: Carresi Nome: ettore
Specie: Cane Razza:
Sesso: Età:
Med. Vet.: Dott.ssa Clara Ferrucci

EMOCROMO

RBC	7.40	5.90 – 8.10	10^6/µl
HGB	17.1	13.1 – 19.0	g/dl
HCT	49.8	36 – 56	%
MCV	67.3	62.0 – 74.0	fL
MCH	23.1	21.0 – 28.0	pg
MCHC	34.3	30.0 – 38.0	g/dl
RDW	12.8	11.5 – 15.9	%
NRBC/100		0 – 0 /100	WBC
WBC	7.16	6.0 – 14.0	10^3/µl
NeuB#		0.0 - 0.3	10^3/µl
NeuS#	6.01	3.62 – 8.9	10^3/µl
**Lym#**	**0.93**	**1.25 – 4.1**	**10^3/µl**
**Mon#**	**0.07**	**0.14 – 0.75**	**10^3/µl**
**Eos#**	**0.14**	**0.15 – 1.16**	**10^3/µl**
Bas#	0.00	0.00 – 0.12	10^3/µl
PLT	179	150 – 460	10^3/µl
MPV	10.2	7.3 – 16.2	fL
PDW	15.3	12.0 – 17.5	%
PCT	0.181	0.09 – 0.500	%

Acantociti
Cheratociti
Codociti
C. di Heinz
C. di H – Jolly
Eccentrociti
Echinociti
Microciti
Macrociti
Schistociti
Sferociti
Sideروciti
Stomatociti

Agglutinazione
Anisocitosi
Ipocromasia
Policromasia
Poichilocitosi
Rouleaux

Linf. Attivi
Linf. Atipici
Linf. Granulari
Neut. Tossici
Granuli tossici
Corpi di Döhle
Basofilia citoplasmatica
Schiuma
citoplasmatica

Aggregati piastrinici
Macropiastrine

Lettura striscio ematico
F. Meduri Med. Vet.

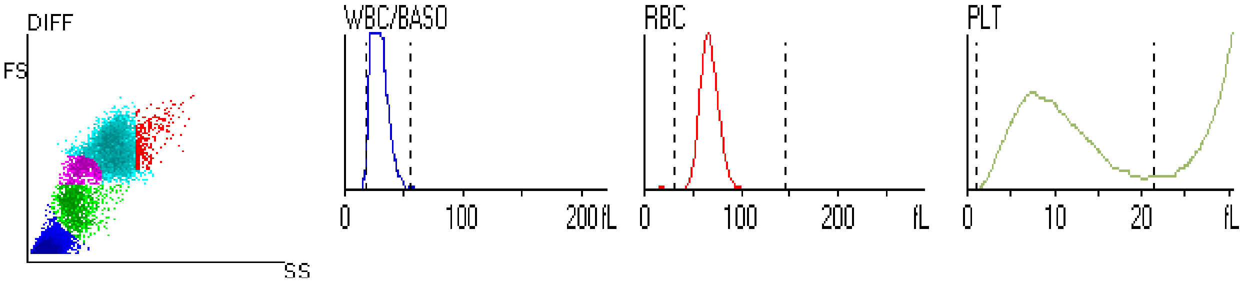

Valori di riferimento validi per la specie canina

Il Direttore Sanitario
Bruno Crucitti Med Vet

NOTE:

Via loreto,99 Reggio Calabria 89133 Tel **0965 594080** E-mail info@bieffelab.it

bieffeLAB
laboratorio di analisi

Reggio Cal 27/09/24

Proprietario: Carresi   Nome: ettore
Specie: Cane   Razza:
Sesso: Età:
Med. Vet.: Dott.ssa Clara Ferrucci

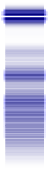

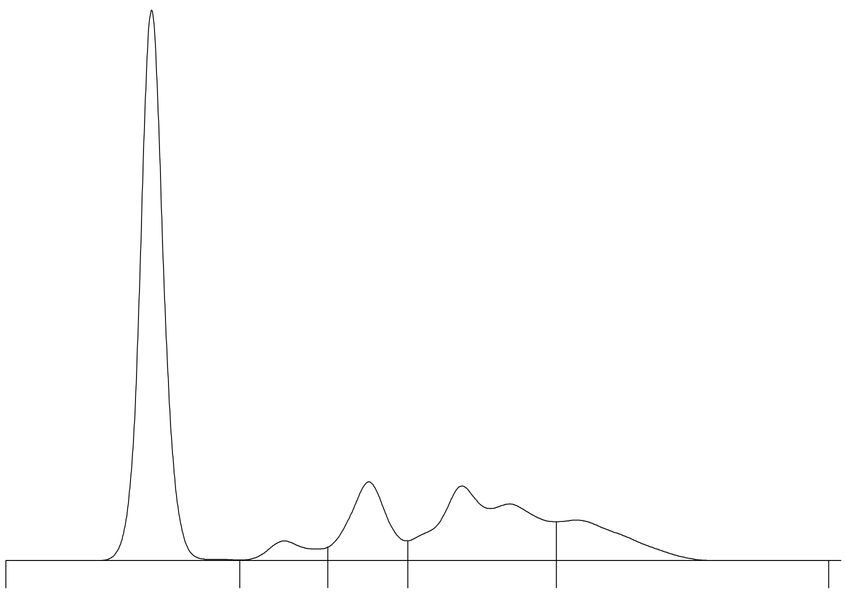

ELETTROFORESI DELLE SIEROPROTEINE

Frazioni		%	Int. Rif.
Prot. Tot.	7.2 g/dl		
Rapp. A/G		1.04	(0.65 – 1.3)
Albumina		51.1	(50.0 – 65.0)
Alfa1		3.3	(2.0 – 6.0)
Alfa2		11.6	(10.0 – 18.5)
**Beta**		**23.6**	**(14.0 – 22.0)**
Gamma		10.4	(6.0 – 15.0)

Il Direttore Sanitario
Bruno Crucitti Med Vet

Via loreto,99 Reggio Calabria 89133 Tel **0965 594080** E-mail info@bieffelab.it
